# Fine‐Tuning of Cholesterol Homeostasis Controls Erythroid Differentiation

**DOI:** 10.1002/advs.202102669

**Published:** 2021-11-05

**Authors:** Zhiyuan Lu, Lixia Huang, Yanxia Li, Yan Xu, Ruihao Zhang, Qian Zhou, Qi Sun, Yi Lu, Junjie Chen, Yuemao Shen, Jian Li, Baobing Zhao

**Affiliations:** ^1^ Key Laboratory of Chemical Biology (Ministry of Education) School of Pharmaceutical Sciences Cheeloo College of Medicine Shandong University Jinan Shandong 250012 China; ^2^ Department of Biochemistry and Molecular Biology School of Basic Medical Sciences Cheeloo College of Medicine Shandong University Jinan Shandong 250012 China; ^3^ Analysis and Measurement Center School of Pharmaceutical Sciences Xiamen University Xiamen Fujian 361001 China; ^4^ Department of Pharmacology School of Pharmaceutical Sciences Cheeloo College of Medicine Shandong University Jinan Shandong 250012 China

**Keywords:** cholesterol homeostasis, enucleation, erythropoiesis, GATA1, ribosome biogenesis, SREBP2

## Abstract

Lipid metabolism is essential for stemness maintenance, self‐renewal, and differentiation of stem cells, however, the regulatory function of cholesterol metabolism in erythroid differentiation is poorly studied. In the present study, a critical role for cholesterol homeostasis in terminal erythropoiesis is uncovered. The master transcriptional factor GATA1 binds to Sterol‐regulatory element binding protein 2 (SREBP2) to downregulate cholesterol biosynthesis, leading to a gradual reduction in intracellular cholesterol levels. It is further shown that reduced cholesterol functions to block erythroid proliferation via the cholesterol/mTORC1/ribosome biogenesis axis, which coordinates cell cycle exit in the late stages of erythroid differentiation. The interaction of GATA1 and SREBP2 also provides a feedback loop for regulating globin expression through the transcriptional control of NFE2 by SREBP2. Importantly, it is shown that disrupting intracellular cholesterol hemostasis resulted in defect of terminal erythroid differentiation in vivo. These findings demonstrate that fine‐tuning of cholesterol homeostasis emerges as a key mechanism for regulating erythropoiesis.

## Graphical abstract

GATA binding protein 1 (GATA1) binds Sterol‐regulatory element binding protein 2 (SREBP2) to downregulate cholesterol biosynthesis, leading to a gradual reduction in cholesterol levels. Lower cholesterol inhibits mTORC1 and ribosome biogenesis and activates p53, thereby coordinating termination of cell cycling in the later stage of erythroid differentiation. Disrupting cholesterol homeostasis interferes with terminal erythropoiesis in vitro and in vivo.

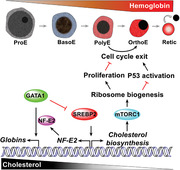

## 1 Introduction

Cholesterol, an essential lipid that localizes predominantly to cell membranes, plays important roles in a diverse array of physiological functions, not only as an important structural component of cell membranes, but also as a precursor of steroid hormones and bile acids. Intracellular cholesterol levels are determined by the dynamic balance between biosynthesis, uptake, export, and esterification.^[^ ^1^ ^]^ These processes are tightly governed by multiple regulatory circuits to ensure cholesterol homeostasis, mediated by the key factors including low‐density lipoprotein receptor (LDLR), transcriptional regulator Sterol‐regulatory element binding protein 2 (SREBP2), acyl coenzyme A: cholesterol acyltransferase (ACAT) and ATP‐binding cassette, sub‐family A (ABC1), ATP binding cassette subfamily G member 1 (ABCG1).^[^ ^1^ ^]^ Disturbed cholesterol balance has been associated with many devastating diseases, such as obesity/diabetes,^[^ ^2^ ^]^ hypercholesterolemia,^[^ ^3^ ^]^ atherosclerosis,^[^ ^4^, ^5^ ^]^ neurodegeneration,^[^ ^6^, ^7^ ^]^ and cancer.^[^ ^8^ ^]^ Recent studies have revealed that metabolic reprogramming is involved in the mammalian erythropoiesis.^[^ ^9^, ^10^, ^11^ ^]^ However, the dynamic changes of lipid metabolism during erythropoiesis is poorly studied.

Mammalian erythropoiesis starts with the commitment of hematopoietic stem cells to erythroid progenitors, including burst forming unit‐erythroid (BFU‐Es) followed by colony forming unit erythroid (CFU‐Es); this is followed by terminal differentiation to give rise to mature red blood cells.^[^ ^12^, ^13^ ^]^ Throughout terminal erythropoiesis, erythroblasts undergo fast cell divisions, cell size decreases, nuclei breakdown and histones releasing, and these events lead to cell cycle exit and nuclear polarization, all required for the final expulsion of the pyknotic nucleus.^[^ ^14^, ^15^, ^16^, ^17^ ^]^ Although multiple molecular and signaling pathways have been revealed to be involved in these processes,^[^ ^18^, ^19^ ^]^ the mechanisms that drive successful maturation and enucleation remain largely undefined. Given that quick cell proliferation of erythroblasts requires cholesterol as building block to support their growth, it is not unreasonable to expect that cholesterol metabolism would play an important role in terminal erythropoiesis.

Multiple lines of evidence support this hypothesis. Mutation of *MVK*, a gene encoding mevalonate kinase in cholesterol biosynthetic pathway, is linked to dyserythropoietic anemia.^[^ ^20^ ^]^ In zebrafish models, cholesterol biosynthesis contributes to the regulation of RBC production.^[^ ^21^, ^22^ ^]^ In addition to cholesterol biosynthesis, mutations of ABCA1 and high‐density lipoprotein receptor SR‐BI resulting in accumulation in erythroblasts, are linked to anemia.^[^ ^23^, ^24^, ^25^ ^]^ Moreover, cyclic changes in cholesterol biosynthesis were observed in stress erythropoiesis induced by phenylhydrazone (PHZ),^[^ ^26^ ^]^ suggesting a possible link between cholesterol levels and erythropoiesis. In line with these studies, a positive association exists between serum cholesterol levels and erythrocyte number.^[^ ^27^, ^28^, ^29^ ^]^ However, the mechanism by which cholesterol regulates terminal erythropoiesis, still remains largely unknown.

In this work, we explored the dynamics of cholesterol homeostasis during terminal erythropoiesis. Interestingly, we found differential requirements of cholesterol during terminal erythropoiesis. The master transcriptional factor GATA1 binds to SREBP2 to downregulate cholesterol biosynthesis, leading to a gradual reduction in intracellular cholesterol levels. More importantly, we demonstrate that reduced cholesterol functions to block erythroid proliferation which coordinates cell cycle exit in the late stages of erythroid differentiation.

## 2 Results

### 2.1 Disruption of Cholesterol Synthesis Impairs the Early Stage Erythroblasts Differentiation

**Figure 1:**
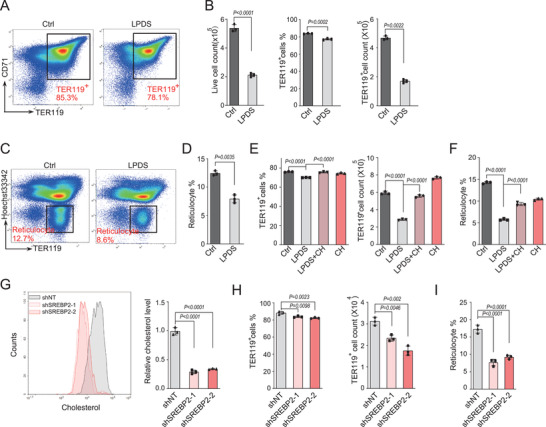
Disruption of cholesterol synthesis impairs the erythroblasts differentiation. A) Representative flow cytometric profiles of erythroid differentiation after in vitro culture with normal FBS (ctrl) or lipoprotein deficient serum (LPDS) for 24 h. B) Statistical analysis of live cells and TER119^+^ erythroid cells in A). C) Representative flow cytometric profiles of erythroid enucleation in cells cultured as in A) for 48 h. D) Statistical analysis of the percentage of reticulocytes in C). E,F) FLCs were cultured in normal FBS or LPDS medium supplemented with or without cholesterol (CH, 10 × 10^−6^ m). Percentage and cell number of TER119^+^ cells (E, 24 h) and reticulocytes (F, 48 h) in total cells were analyzed by flow cytometry. G–I) FLCs were transduced with retroviruses encoding indicated shRNA and cultured in SCF medium for 12 h, and then changed into Epo medium. Intracellular cholesterol levels G, 24 h), percentage and cell number of TER119^+^ erythroid cells(H, 24 h) and percentage of reticulocytes in cells cultured for 48 h I, 48 h) were quantified by flow cytometric analysis. shNT: Scramble shRNA. *P* values were determined by using unpaired two‐tailed Student's *t*‐test B,D) or 1‐way ANOVA with Tukey's multiple comparisons test E–I). Data are presented as mean ± SD from three independent experiments. See also Figure S1 (Supporting Information).

To investigate the roles of cholesterol homeostasis in terminal erythropoiesis, we used a well‐established ex vivo mouse fetal liver cell culture system, in which TER119‐negative erythroblasts isolated from E14.5 fetal liver (FLCs) were differentiated into enucleated reticulocytes in vitro.^[^ ^30^, ^31^ ^]^ We first cultured the erythroid progenitors with lipoprotein deficient serum (LPDS) to block exogenous cholesterol uptake. Relative to normal culture, cell percentage and number of TER119^+^ erythroblasts were significantly lower in LPDS group after 1‐day of culture (**Figure** **1**A,B). The LPDS group also exhibited a lower extent of enucleation compared with the control group (Figure 1C,D). Similar repression of erythroid differentiation was also observed in erythroblasts treated with U18666A,^[^ ^32^ ^]^ a cholesterol transport inhibitor (Figure S1A,B, Supporting Information). Importantly, the inhibition of cell proliferation and differentiation, as well as enucleation in the LPDS group was greatly rescued by supplementation of cholesterol, suggesting that the blockade of erythroid proliferation and differentiation by LPDS was at least partially caused by limiting cellular cholesterol uptake (Figure 1E,F).

We next treated the erythroblasts with fatostatin that targets maturation of SREBP2 to inhibit intracellular cholesterol biosynthesis.^[^ ^33^ ^]^ TER119^+^ erythroblasts cell percentage and cell number were significantly reduced after 1‐day of culture with fatostatin (Figure S1C, Supporting Information), followed by a lower extent of enucleation compared with control cells (Figure S1D, Supporting Information). As expected, supplementation of cholesterol markedly relieved the repressed cell proliferation and differentiation induced by fatostatin (Figure S1E, Supporting Information). Consistently, lovastatin^[^ ^34^ ^]^ and YM53601,^[^ ^35^ ^]^ targeting HMGCR and SQLE, respectively, two key enzymes of the cholesterol biosynthesis, also substantially inhibited the erythroblasts proliferation and differentiation (Figure S1F–H, Supporting Information). However, inhibition of cholesterol biosynthesis or exogenous cholesterol uptake had no detectable effect on the cell proliferation and enucleation when TER119‐negative fetal liver cells were treated after 30 h of in vitro culture in Epo medium, corresponding to the late stages of terminal erythroid differentiation (Figure S1I–P, Supporting Information).

In addition to the pharmacological approaches, we employed a genetic tool to abrogate SREBP2 expression using shRNA in FLCs (Figure S1Q, Supporting Information). The intracellular cholesterol levels were significantly reduced in SREBP2‐knockdown erythroblasts compared with control cells (Figure 1G). Again, knockdown of SREBP2 also resulted in defects of erythroid differentiation and terminal enucleation (Figure 1H,I). These data demonstrated that cholesterol is critical for the early stage erythroblasts proliferation and differentiation.

### 2.2 Excess Cholesterol Impairs Terminal Erythropoiesis by Disrupting Cell Cycle Exit and Terminal Enucleation

**Figure 2:**
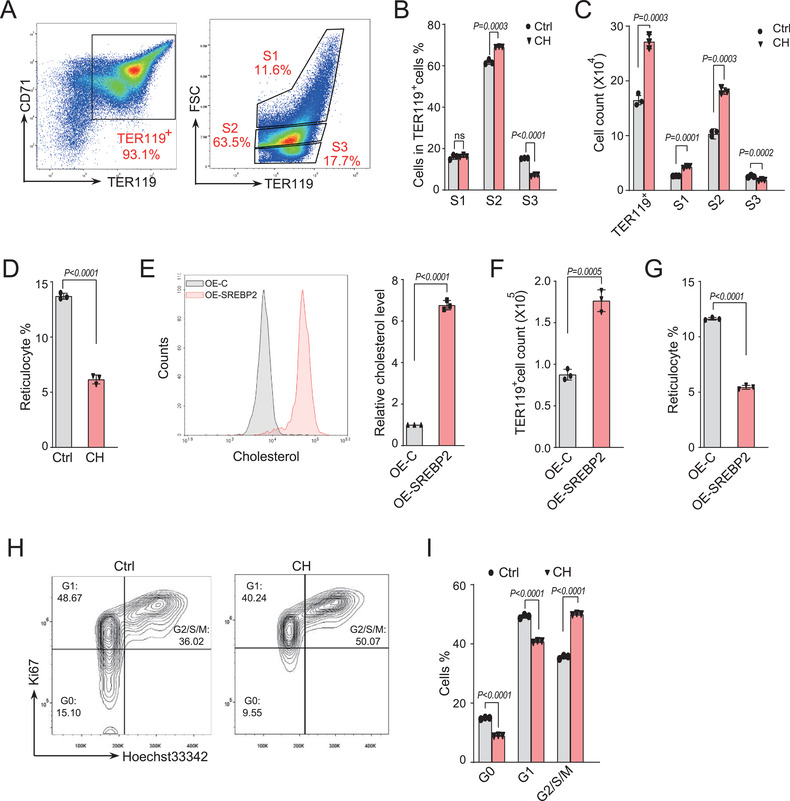
Excess cholesterol impairs terminal erythropoiesis by disrupting cell cycle exit and terminal enucleation. A) Representative flow cytometric profiles of FLCs after 48 h of in vitro culture. TER119^+^ cells were subdivided into S1, S2, and S3 based on the forward scatter (FSC). B,C) Statistical analysis of percentage and cell number of TER119^+^ cells in indicated S population in A) with treatment of cholesterol (40 × 10^−6^ m) for 48 h. Ctrl: vehicle control; CH: cholesterol. D) Statistical analysis of the percentage of reticulocytes in total cells after treatment as in A) for 48 h. E) FLCs were transduced with retroviruses encoding active SREBP2 (1‐468aa) or blank vector, and then cultured in SCF medium for 12 h followed by Epo medium culture for 24 h, intracellular cholesterol levels were measured (left) and quantified (right) by flow cytometry using Filipin III staining. F,‐G) Cells were transduced as in E) and then cultured in Epo medium for 48 h, cell number of TER119^+^ cells F) and percentage of reticulocytes G) were quantified by flow cytometry. H) Representative cell cycle profile of erythroblasts after in vitro culture with or without cholesterol (40 × 10^−6^ m) for 36 h. Cell cycle was analyzed by flow cytometry using Hoechst 33342 and Ki67 staining. Ctrl: vehicle control; CH: cholesterol. The percentage of cells in each phase is shown. I) Quantification of cells in indicated phases in H). All *P* values were determined by unpaired two‐tailed Student's *t*‐test. Data are presented as mean ± SD from three independent experiments. See also Figure S2 (Supporting Information).

Most TER119‐negative erythroid cells were differentiated into TER119^+^ cells after 2 days of culture, which was also observed in exogenous cholesterol treated cells (data not shown). To further dissect the effect of cholesterol on the late stages of terminal erythroid differentiation, we used forward scatter to divide TER119^+^ erythroid cells into 3 populations (S1–S3) since cell size is known to gradually decrease during erythroid terminal maturation (**Figure** **2**A). The percentage and cell number of S1 and S2 erythroid cells were significantly increased after cholesterol treatment that accounted for a dramatic increase in total TER119^+^ erythroid cell number, while that of S3 erythroid cells that are mainly reticulocytes were markedly reduced (Figure 2B,C). This is consistent with a significant inhibition of enucleation in cells with cholesterol treatment (Figure 2D). Morphological analyses also showed a decreased number of enucleated reticulocytes and enlarged nuclei in cholesterol treated erythroid cells (Figure S2A, Supporting Information). Moreover, similar defects of erythroblast maturation were also observed when TER119‐negative fetal liver cells were treated with excess cholesterol after 30 h of in vitro culture, corresponding to the late stages of terminal erythroid differentiation (Figure S2B, Supporting Information).

In addition to exogenous cholesterol uptake, the intracellular cholesterol level is also regulated by the biosynthesis, which is controlled by the master transcriptional factor SREBP2. To further confirm the importance of reduced cholesterol on the terminal erythropoiesis, we forced to elevate the endogenous cholesterol level in FLCs through overexpression of activated SREBP2 (1‐468aa) (Figure S2C, Supporting Information). Flow cytometric analysis demonstrated that the intracellular cholesterol levels were dramatically increased in erythroblasts overexpressing SREBP2 (Figure 2E). SREBP2 overexpression enhanced erythroblast proliferation (Figure 2F), and repressed subsequent terminal enucleation (Figure 2G). These findings indicate that excess cholesterol promote erythroid cells proliferation that contribute to a defect of erythroblast maturation at late stages.

Given that continued cell cycling at the late stage of erythroid terminal differentiation results in an enucleation defect,^[^ ^15^, ^36^, ^37^ ^]^ we reasoned that continuous cell proliferation by forced increase of cholesterol would impair the cell cycle exit, which in turn would lead to a defect in the ability of orthochromatic erythroblasts to enucleate. To test our hypothesis, we analyzed the cell cycle status of erythroid cells cultured for 36 h in vitro, when a majority of cells are differentiated into orthochromatic erythroblasts stage.^[^ ^38^ ^]^ More cells stayed at the G2/S/M‐phase after cholesterol treatment, while cells at G0/G1 phase were significantly reduced (Figure S2D,E, Supporting Information). On the other hand, depletion of cellular cholesterol by LPDS treatment led to an increase of cells at G0/G1 and a reduction of those at the G2/S/M‐phase (Figure S2F,G, Supporting Information).

To further test whether cell‐cycle exit was affected by increased cholesterol, erythroid cells were cultured in Epo medium for 36 h and stained for Ki67, a cell proliferation marker that is specifically expressed in cycling cells (Figure 2H). The percentage of cells in G0‐phase was dramatically decreased after cholesterol treatment, indicating that fewer cells exited from cell cycle (Figure 2I). These data suggest that reduced cholesterol is required for the cell cycle exit at the late stages of terminal differentiation.

### 2.3 Cholesterol Regulates Erythropoiesis by Controlling Ribosome Synthesis

**Figure 3:**
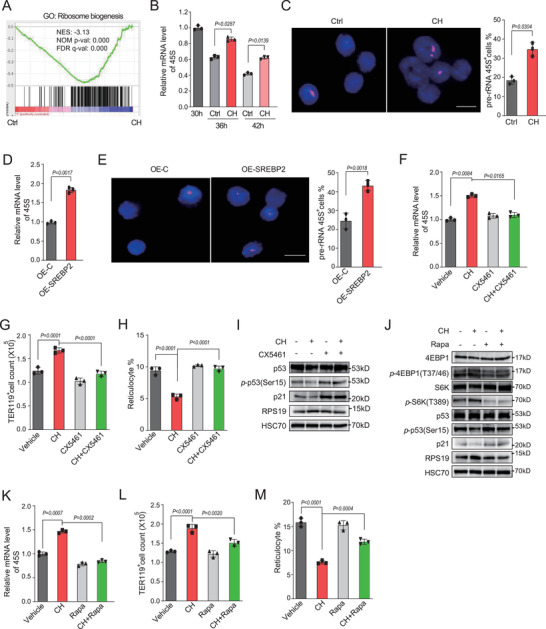
Cholesterol regulates erythropoiesis by controlling ribosome biogenesis. A) Gene Set Enrichment Analysis of ribosome biogenesis pathway in erythroblasts treated by cholesterol (CH) or vehicle (control, Ctrl). FLCs were cultured in Epo medium for 30 h supplemented with or without cholesterol (40 × 10^−6^ m), and then TER119^+^ cells were sorted and subjected to RNA deep sequencing. B) Quantification of pre‐rRNA 45S transcripts in erythroblasts with treatment of cholesterol. FLCs were cultured in Epo medium for 30 h, and then treated with cholesterol for additional 6 or 12 h. C) Fluorescence in situ hybridization for pre‐rRNA 45S in indicated cells from B) (left) and statistical analysis of percentage of pre‐rRNA 45S fluorescence positive cells (right). Scale bars, 5 µm. D) Quantification of transcripts of pre‐rRNA 45S in erythroblasts with SREBP2 overexpression. FLCs were transduced with retroviruses encoding active SREBP2 (1‐468aa) and cultured in Epo medium for 42 h. E) Fluorescence in situ hybridization for pre‐rRNA 45S in indicated cells from D) (left) and statistical analysis of percentage of pre‐rRNA 45S fluorescence positive cells (right). Scale bars, 5 µm. F–H) Quantification of mRNA expression of pre‐rRNA 45S F), TER119^+^ cells number G), and percentage of reticulocytes H) in erythroblasts cultured in Epo medium for 30 h, followed by treatment with or without CX5461(50 × 10^−9^ m) for 12 h in the presence of cholesterol (CH). I) Western blot analysis of indicated proteins in cells from F). HSC70 was used as a loading control. An equal number of cells were loaded in each well. J–M) Cells were treated as in F–H), except that cells were treated with or without rapamycin (RAM) (100 × 10^−9^ m). Indicated proteins levels were analyzed by Immunoblotting J). HSC70 was used as a loading control. An equal number of cells were loaded in each well. The mRNA expression of pre‐rRNA 45S K), cell number of TER119^+^ cells L), and percentage of reticulocytes M) were quantified. *P* values were determined by using unpaired two‐tailed Student's *t*‐test B–E) or 1‐way ANOVA with Tukey's multiple comparisons test F, G, H, K, L, M). Data are presented as mean ± SD from three independent experiments. See also Figure S3 (Supporting Information).

To understand the molecular mechanism by which cholesterol promotes erythroid proliferation and blocks cell cycle exit, TER119^+^ erythroblasts were sorted and subjected to RNA‐seq analysis after 30 h of in vitro culture in the presence or absence of cholesterol. As expected, Gene Set Enrichment Analysis showed a down‐regulation of cholesterol/steroid biosynthetic pathway (Figure S3A and Table S1, Supporting Information) by cholesterol treatment, reflecting feedback regulation of excess cholesterol on cholesterol biosynthesis. Strikingly, gene cluster of ribosome biogenesis was highly enriched and upregulated after cholesterol treatment (**Figure** **3**A; and Table S2, Supporting Information). Ribosome biogenesis starts in the nucleolus by the transcription of ribosomal DNA into a 45S rRNA precursor by RNA polymerase (*pol*)I. Indeed, 45S rRNA precursor expression exhibited a sharp decrease in normal differentiating erythroblasts at day 2 in vitro culture that corresponds to the late stages of terminal erythropoiesis in vivo (Figure S3B,C, Supporting Information).

In line with the enrichment of gene cluster of ribosome biogenesis, we found that cholesterol significantly increased the level of 45S rRNA that was decreased in normal erythroblasts differentiation (Figure 3B), which was further confirmed by fluorescence in situ hybridization (Figure 3C). The ribosome profile showed that the stoichiometry of free 40S and 60S ribosomal subunits, assembled 80S and polysomes was mostly unchanged, however, all of them exhibited a mild increase after 12 h of treatment by cholesterol (Figure S3D, Supporting Information). Similar upregulation of 45S rRNA transcription was also observed in erythroblasts overexpressing SREBP2 (Figure 3D,E). These data indicated that excess cholesterol enhances the ribosome biogenesis during terminal erythroid maturation.

To better understand the role of ribosome biogenesis in defective terminal erythroid differentiation caused by excess cholesterol, we treated erythroblasts with CX5461,^[^ ^39^ ^]^ a specific RNA *pol*I inhibitor, after the cells were cultured for 30 h and differentiated into late stages of terminal erythroid differentiation. As expected, the increased transcription of 45S rRNA by cholesterol was normalized by CX5461 (Figure 3F). Importantly, CX5461 completely abrogated the effect of cholesterol on erythroid differentiation, including promoting cell proliferation and impaired final enucleation (Figure 3G,H).

P53 activation and cell cycle arrest coincides with altered ribosome biogenesis in normal erythroid differentiation and ribosomopathies.^[^ ^40^ ^]^ Indeed, cell cycle genes were highly enriched after cholesterol treatment (Figure S3E, Supporting Information). Western blotting analysis demonstrated that cholesterol treatment reduced the phosphorylation of p53 and its transcriptional target p21. Strikingly, CX5461 completely blocked the effect of cholesterol on the phosphorylation of p53 as well as p21 protein levels (Figure 3I; and Figure S3F, Supporting Information), indicating that disrupted ribosome biogenesis by cholesterol overload contributes to continuous cell cycling upon the p53 inactivation.

The mechanistic target of rapamycin complex 1 (mTORC1) protein kinase is a master growth regulator that can be activated by cholesterol,^[^ ^41^ ^]^ and mTORC1 activation leads to a boost of ribosome biogenesis.^[^ ^42^ ^]^ We therefore investigated the role of the cholesterol/mTOCR1/ribosome biogenesis axis in terminal erythropoiesis. Cholesterol showed a mild but consistent effect on mTORC1 activation indicated by increased phosphorylation of 4EBP1 and S6K, as well as p53 inactivation, which was largely abolished by mTORC1 inhibitor rapamycin^[^ ^43^ ^]^ (Figure 3J; and Figure S3G, Supporting Information). Moreover, cholesterol reversed the mTORC1 activity repressed by LPDS treatment (Figure S3H, Supporting Information). The critical role of mTORC1 in the regulation of erythroid differentiation was further supported by the observation that rapamycin rescued the defect of terminal erythropoiesis induced by excess cholesterol (Figure 3K–M). Together, these data suggest that activation of mTORC1 by cholesterol dictates the up‐regulation of ribosome biogenesis and subsequent p53 inactivation, leading to the impairment of erythroid maturation.

### 2.4 Cholesterol Synthesis is Down‐Regulated During Terminal Erythropoiesis

Given that early and late stages of terminal erythropoiesis have distinct requirements on cellular cholesterol levels, we next examined the dynamic changes of cholesterol during erythroid differentiation. Instead of investigation of in vitro culture of fetal liver cells, in which the erythroid cells are not synchronously differentiated and possibly contaminated by nonerythroid cells, we used the well‐studied erythroid K562 cell line.^[^ ^30^ ^]^

**Figure 4:**
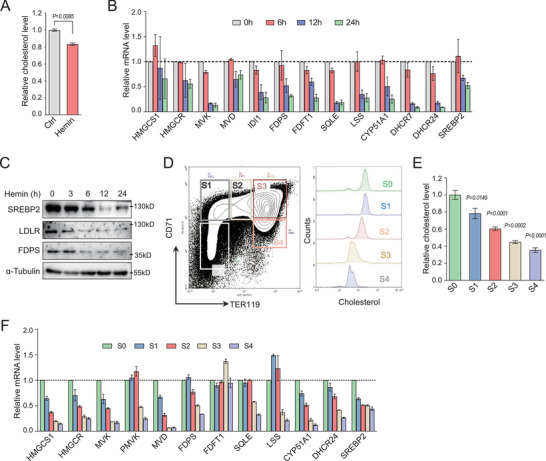
Cholesterol synthesis is down‐regulated during terminal erythropoiesis. A) Quantification of intracellular cholesterol in K562 cells after treatment with vehicle or hemin (40 × 10^−6^ m) for 24 h. *P* values were determined by using unpaired twotailed Student's *t*‐test. B) Quantification of mRNA expression of indicated genes in K562 cells after incubation of hemin. C) Immunoblot analysis of indicated proteins in K562 cells after induction of hemin for indicated time. *α*‐tubulin was used as a loading control. An equal number of cells were loaded in each well. D) Representative flow cytometric profiles of E14.5 mouse fetal liver cells stained with CD71 and TER119 antibodies. The S0–S4 populations were gated based on the protein levels of CD71 and TER119 (left). Intracellular cholesterol levels were analyzed by flow cytometry based on the intensity of Filipin III (50 µg mL^−1^) staining of S0–S4 populations (right). E) Quantification of relative cholesterol levels that normalized to phosphatidylserine level in indicated cells in D). *P* values were determined by using 1‐way ANOVA with Tukey's multiple comparisons test. F) Quantitative PCR analysis of the mRNA level of genes encoding cholesterologenic enzymes. Cells were sorted by flow cytometry according to D). Data are presented as mean ± SD from three independent experiments. See also Figure S4 (Supporting Information).

Interestingly, the cellular level of cholesterol was significantly reduced in erythroid‐differentiated K562 cells with hemin stimulation (**Figure** **4**A). Intracellular cholesterol levels could be controlled by biosynthesis, uptake, export, and esterification. However, the mRNA level of export and esterification related genes were downregulated during terminal erythropoiesis, including ABCA1/ABCG1 and ACAT1/ACAT2 (Figure S4A, Supporting Information). Genes encoding cholesterologenic enzymes were transcriptionally down‐regulated during differentiation of K562 induced by hemin (Figure 4B; and Figure S4B, Supporting Information). Accordingly, the protein levels of SREBP2 and FDPS that represent cholesterol biosynthetic pathway were also reduced in these cells (Figure 4C). LDLR, an important receptor for exogenous cholesterol uptake and also a target of SREBP2, was also downregulated during differentiation of K562 induced by hemin (Figure 4C). However, hemin treatment induced a marginal increase of these transcripts in HeLa cells (Figure S4C, Supporting Information), which suggested that hemin initiates the erythroid differentiation pathway to control cholesterol biosynthesis. Consistently, repressed transcription of cholesterol synthesis‐related enzymes was also observed in K562 cells induced by butyrate, albeit to a lesser extent (Figure S4D, Supporting Information).

We further investigated the dynamics of cholesterol in in vivo erythropoiesis. Five subpopulations of erythroblasts (S0–S4) were identified from E14.5 mouse fetal livers according to the surface expression of CD71 and TER119, representing a sequence of erythroblast differentiation (Figure 4D).^[^ ^44^ ^]^ Considering that reduced cell size may result in the decrease of the contents of various membrane lipids during erythroblast differentiation, we normalized the cellular cholesterol to that of intracellular phosphatidylserine, and found that the amount of cholesterol was dramatically decreased from the S0 to S4 (Figure 4E; and Figure S4E, Supporting Information). Cholesterologenic enzymes were also transcriptionally down‐regulated (Figure 4F), consistent with our observations in K562 cells. Similar transcriptional change was also observed during terminal erythroid differentiation in adult mouse bone marrow (Figure S4F,G, Supporting Information). Altogether, these observations demonstrate that the gradually reduction in cholesterol is correlated with down‐regulated cholesterol biosynthesis during terminal erythropoiesis.

### 2.5 GATA1 Downregulates Cholesterol Biosynthesis by Interacting with SREBP2

**Figure 5:**
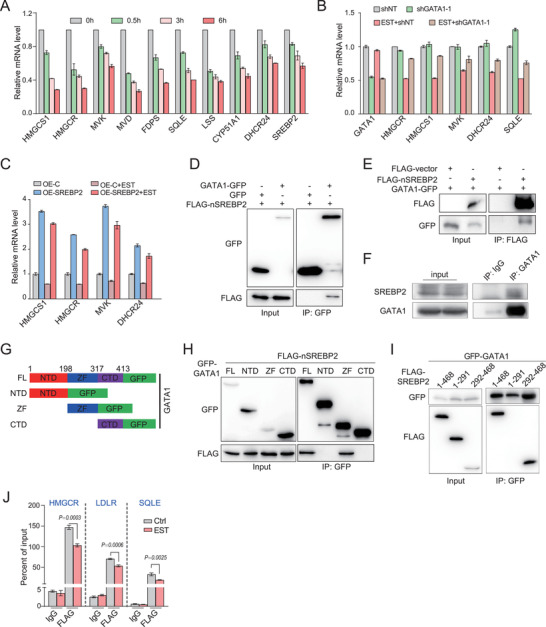
GATA1 downregulates cholesterol biosynthesis by interacting with SREBP2. A) Quantification of mRNA expression of indicated genes in G1‐ER4 cells after induction of *β*‐estradiol (EST, 500 × 10^−9^ m). B,C) Quantification of mRNA expression of indicated genes in G1‐ER4 cells with GATA1 knockdown or SREBP2 overexpression. G1‐ER4 cells were transduced with retroviruses encoding GATA1 shRNA B) or active SREBP2 (1‐468aa) C), and then induced by EST for 3 h. shNT: Scramble shRNA. D,E) Reciprocal Co‐IP of exogenous FLAG‐nSREBP2 and GFP‐GATA1 in 293T cells. F) Co‐IP of endogenous SREBP2 and GATA1 in G1ER cells after induction of *β*‐estradiol. G) Structure of full‐length and truncated GATA1. FL, full length; NTD, N‐terminal domain; ZF, zinc finger; CTD, C‐terminal domain. H) Co‐IP assay of truncated GATA1 and FLAG‐nSREBP2 (1‐468aa). I) Co‐IP assay of truncated FLAG‐nSREBP2 and GATA1. J) Chromatin Immuno‐Precipitation analysis of promoter binding activity of SREBP2 to its target genes in cells from C). All *P* values were determined by unpaired two‐tailed Student's *t*‐test. Data are presented as mean ± SD from three independent experiments. See also Figure S5 (Supporting Information).

To determine whether the master transcription factor GATA1 is involved in the regulation of cholesterol biosynthesis in erythropoiesis, we used a well‐studied erythroid G1E‐ER4 cell line, which stably expresses murine GATA1 fused to the ligand‐binding domain of the human estrogen receptor. Upon supplementation with *β*‐estradiol (EST) which restores GATA1 transcriptional activity, this cell line closely mimics the primary erythroid differentiation process.^[^ ^45^ ^]^ qPCR analysis showed that the expression of genes encoding cholesterologenic enzymes was gradually decreased in G1E‐ER4 cells after *β*‐estradiol induction (**Figure** **5**A; and Figure S5A, Supporting Information), consistent with our observations in K562 cells and mouse erythroblasts (Figure 4). To rule out the possibility that *β*‐estradiol down‐regulates transcription of cholesterol biosynthetic genes independent of GATA1, G1E‐ER4 cells were transduced with GATA1 shRNA. Transcription of cholesterol biosynthetic genes exhibited no difference in GATA1 knockdown cells in which GATA1 is in an inactivated state in the absence of *β*‐estradiol. In contrast, decreased expression of these genes in *β*‐estradiol treated cells were notably rescued by GATA1 knockdown (Figure 5B; and Figure S5B, Supporting Information). In line with this, GATA1 knockdown in K562 cells also led to increased expression of these genes, further confirming a direct role of GATA1 in the regulation of cholesterol biosynthesis (Figure S5C, Supporting Information).

SREBP2 is proteolytically cleaved at the Golgi, to release a fragment that binds to the promoters of target genes to upregulate cholesterol biosynthesis.^[^ ^46^, ^47^ ^]^ Blockade of SREBP2 activation could result in decreased expression of cholesterol synthesis related genes. However, SREBP2 cleavage was not inhibited when K562 cell differentiation was induced by hemin (Figure S5D, Supporting Information), suggesting otherwise. Instead, rapid downregulation of most cholesterol biosynthesis related genes in G1E‐ER4 cells after 0.5 h *β*‐estradiol induction, strongly suggested that GATA1 directly affects the transcriptional activity of SREBP2 (Figure 5A). Interestingly, overexpression of proteolytically activated nSREBP2 (1‐468aa) upregulated cholesterologenic enzymes expression in G1E‐ER4 cells, which barely responded even to *β*‐estradiol induction (Figure 5C; and Figure S5E, Supporting Information), suggesting that excess nSREBP2 attenuated the inhibitory effect of GATA1.

We therefore investigated whether SREBP2 physically interacts with GATA1. SREBP2 and GATA1 were reciprocally coimmunoprecipitated with each other, indicating a physical interaction (Figure 5D,E). This is further confirmed by the co‐IP assays of endogenous GATA1 and mature SREBP2 in G1‐ER4 and K562 cells (Figure 5F; and Figure S5F, Supporting Information). To map the domains that are critical for the physical interaction of GATA1 and SREBP2, we constructed a series of truncated forms of the two proteins (Figure 5G; and Figure S5G, Supporting Information). Strikingly, SREBP2 (292–468 aa) showed specific interaction with zinc‐finger domains of GATA1 by co‐IP assay (Figure 5H,I).

We then evaluated the effect of GATA1 on the SREBP2 transcriptional activity. Chromatin immunoprecipitation (ChIP) followed by qPCR was performed to monitor the chromatin occupancy of SREBP2 on its target genes in G1E‐ER4 cells. As expected, SREBP2 exhibited notable recruitment to the promoters of *HMGCR*, *LDLR*, and *SQLE*. Importantly, SREBP2 chromatin occupancy at these sites was significantly decreased upon the GATA1 activation induced by *β*‐estradiol (Figure 5J). These data demonstrated that GATA1 interacts with and represses the transcriptional activity of SREBP2, to downregulate cholesterol biosynthesis.

### 2.6 Transcriptional Control of NFE2 by SREBP2 Contributes to the Regulation of Globin Expression

**Figure 6:**
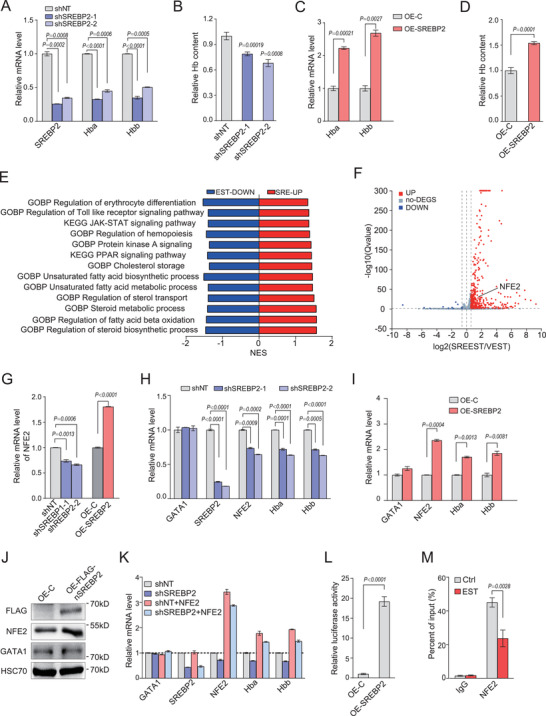
Transcriptional control of NFE2 by SREBP2 contributes to the regulation of globin expression. A) Quantification of mRNA expression of *SREBP2*, *Hba*, and *Hbb* in erythroblasts with SREBP2 knockdown. FLCs were transduced with retrovirus encoding SREBP2 shRNA and cultured in SCF medium for 12 h, and then changed into Epo medium for additional 24 h. B) Quantification of hemoglobin content in indicated cells from A). C) Quantification of mRNA expression of *Hba* and *Hbb* in erythroblasts with SREBP2 overexpression. FLCs were transduced with retrovirus encoding active SREBP2 (1‐468aa) and cultured as in A). D) Quantification of hemoglobin content in indicated cells from C). E,F) Global transcriptome analysis were performed in which EST stimulated G1E‐ER4 cells were overexpressed with mature SREBP2 or blank vector. Representative GO_BP terms and KEGG pathways ranked by NES values were shown in E) from GSEA analysis (FDR<0.25, *P* value <0.05), and the total list of enriched GO_BP terms and KEGG pathways are shown in Tables S4 and S5 (Supporting Information). UP: upregulated in SREBP2 overexpression cells with EST treatment; DOWN: downregulated in blank vector cells with EST treatment. F) The volcano plot of the differentially expressed genes between SREBP2 overexpression with blank vector stimulated by EST (fold change >1.5 and *P* value <0.05). G) Quantification of mRNA expression of *NFE2* in cells from A) and C). H,I) Quantification of mRNA expression of indicated genes in G1‐ER4 cells with knockdown or overexpression of activated SREBP2 (1‐468aa). shNT: Scramble shRNA. J) Immunoblot analysis of indicated proteins in cells from G). HSC70 was used as a loading control. An equal number of cells were loaded in each well. K) Quantification of mRNA expression of indicated genes in G1‐ER4 cells with SREBP2 shRNA and NFE2 overexpression. L) Luciferase reporter assay of *NFE2* promoter. HEK293T cells were cotransfected with mouse NFE2 luciferase reporter constructs in the present of FLAG‐SREBP2 (1‐468aa) expressing plasmid or blank vector. M) Chromatin Immuno‐Precipitation analysis of promoter binding activity of SREBP2 to *NFE2* in G1‐ER4 cells treated as in Figure 5J. All *P* values were determined by unpaired two‐tailed Student's *t*‐test. Data are presented as mean ± SD from three independent experiments. See also Figure S6 (Supporting Information).

We assessed whether the interaction of GATA1 with SREBP2 also affects the transcriptional activity of GATA1, characterized by GATA1 target gene expression, including itself and well‐known globin genes. Neither overexpression nor knockdown of SREBP2 in mouse fetal erythroid progenitors had obvious effects on GATA1 mRNA levels (data not shown). In contrast, globin gene transcription was increased upon SREBP2 overexpression, but down‐regulated by depletion of SREBP2 (**Figure** **6**A,C). This was further confirmed by quantitative analysis of hemoglobin content in erythroblasts upon overexpression or knockdown of SREBP2, respectively (Figure 6B,D).

To better understand the consequences of the interaction of SREBP2 and GATA1, we analyzed the global transcriptomic profiles of G1E‐ER4 cells with SREBP2 overexpression or EST stimulation, respectively, compared with that of blank vector control. As expected, cholesterol biosynthesis related‐genes and GATA1 target genes are significantly regulated after SREBP2 overexpression and stimulation, respectively (Figure S6A,B, Supporting Information). Surprisingly, the subsequent differential expression analysis showed that 4979 (72.8%) differential genes were shared by SREBP2 overexpression and EST treatment (1.2‐fold change cutoff) (Figure S6C, Supporting Information). Among these overlapped genes, only 80 (1.6%) genes were oppositely regulated (significantly upregulated by SREBP2 but showed down‐regulation in cells with EST treatment) (Figure S6C, Supporting Information). However, DAVID analysis of genes oppositely regulated in both groups with onefold change cutoff indicated that erythroid differentiation and lipid metabolic process, as well as cholesterol biosynthesis, were among the highly enriched GO_BP terms and Kyoto Encyclopedia of Genes and Genomes (KEGG) pathways (*P* value < 0.05, FDR< 25%) (Table S3, Supporting Information).

The fact that overexpression of SREBP2 largely mimics GATA1 activation led us to hypothesize that overexpressed SREBP2 would bind GATA1 and bring it to nucleus for trans‐activation. Indeed, we found that SREBP2 overexpression induced nuclear localization of the GATA1/ER proteins which mainly distributed in the cytoplasm of G1E‐ER4 in the absence of EST (Figure S6D, Supporting Information). Given that GATA1 interacts with and in turn represses the transcriptional activity of SREBP2, the effects of SREBP2 overexpression on the target genes may be compromised due to the activation of GATA1 induced by its overexpression. To further estimate the overlap of GATA1 and SREBP2 transcriptional targets, we performed another global transcriptome analysis in which EST stimulated G1E‐ER4 cells were overexpressed with SREBP2 or blank vector. GSEA of the transcriptome revealed numerous highly enriched GO_BP terms and KEGG pathways that were also shown in the enrichments of the gene sets from single EST treatment, including Janus kinases (JAK)/Signal transducer and activator of transcription (STAT) signaling, peroxisome proliferators‐activated receptor (PPAR) signaling, erythroid differentiation and lipid and cholesterol metabolism, suggesting that SREBP2 mediated the regulation of GATA1 on these biological processes and signaling pathways during erythroid differentiation (Figure 6E; and Tables S4 and S5, Supporting Information).

The subsequent differential expression analysis showed that 490 genes were significantly upregulated by the SREBP2 overexpression compared to that of cells transduced with blank vector upon EST stimulation (>1.5‐fold change) (Figure 6F). Among these genes, nuclear factor erythroid 2 (NFE2) is a transcription factor that cooperates with GATA1 to regulate globin gene expression.^[^ ^48^ ^]^ qPCR analysis confirmed that NFE2 mRNA level was positively regulated by SREBP2 in fetal liver erythroblasts (Figure 6G). Similar transcriptional profiles of NFE2 as well as globin genes were also observed in G1‐ER4 cells (Figure 6H,I; and Figure S6E, Supporting Information). The level of NFE2 protein but not GATA1 was also upregulated in G1E‐ER4 cells overexpressing SREBP2, compared with that of control cells (Figure 6J). Furthermore, NFE2 overexpression fully restored globin gene expression that was downregulated in G1E‐ER4 cells upon SREBP2 knockdown (Figure 6K). The transcription level of NFE2 positively correlates with that of globin genes, suggesting that the regulation of SREBP2 on the globin genes expression is dependent on NFE2.

To determine whether SREBP2 directly control NFE2 transcription, we performed a luciferase reporter assay with *NFE2* promoter. SREBP2 overexpression resulted in a marked increase of luciferase activity in 293T cells transduced with *NFE2* promoter (Figure 6L). We further analyzed chromatin occupancy of SREBP2 on the NFE2 promotor region in G1E‐ER4 cells by ChIP followed by qPCR. The ability of SREBP2 to bind to the *NFE2* promoter was significantly reduced upon inhibition of SREBP2 transcriptional activity by *β*‐estradiol‐induced GATA1 activation (Figure 6M). Collectively, these data demonstrate that SREBP2 regulates globin expression via transcriptional regulation of NFE2.

### 2.7 Excess Cholesterol Impairs Erythropoiesis In Vivo

**Figure 7:**
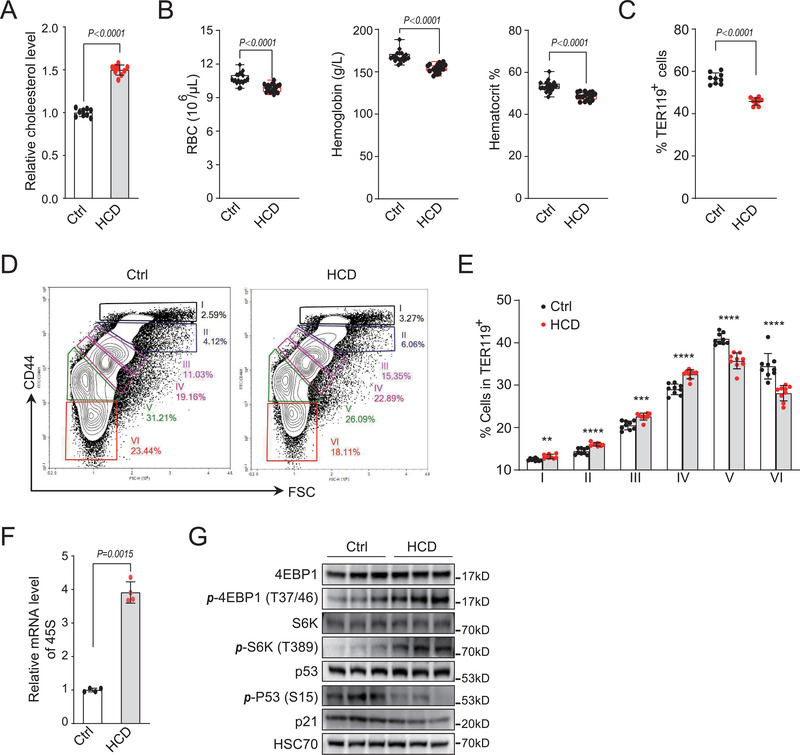
Excess cholesterol impairs erythropoiesis in vivo. A) Quantification of serum cholesterol in high cholesterol diet fed mice. C57BL/6J mice were fed on chow diets (*n* = 9) or high cholesterol diets (HCD) (*n *= 9) for 4 weeks. Both males and females were included in each group. B) Red blood cells (RBC), hemoglobin and hematocrit indices of indicated mice. Each dot represents one mouse. C) Quantification of percentage of TER119^+^ cells in bone marrow from indicated mice. Each dot represents one mouse. D) Representative flow cytometric profiles of erythroid populations based on CD44 and TER119 expression from C). Populations I to VI represent the least differentiated to enucleated RBC. E) Quantification of percentage of indicated populations from D). *P < 0.05, **P < 0.01, ***P < 0.001, and ****P < 0.0001. F) Quantification of transcripts of pre‐rRNA 45S in erythroblasts from indicated mice bone marrow in A). G) Western blot analysis of indicated proteins in erythroblasts from indicated mice bone marrow in A). HSC70 was used as a loading control. An equal number of cells were loaded in each well. All *P* values were determined by unpaired two‐tailed Student's *t*‐test. See also Figures S7 and S8 (Supporting Information).

To further confirm the importance of cholesterol homeostasis in erythropoiesis in vivo, we first put mice on a high‐cholesterol diet (HCD) for 4 weeks. HCD feeding significantly increased the serum concentration of total cholesterol (**Figure** **7**A). Compared with chow diet‐fed mice, the HCD group mice showed significantly lower red blood cell number (RBC), hemoglobin level and hematocrit (Figure 7B), while they exhibited a mild increase in white blood cell count and no difference in platelet numbers (Figure S7A, Supporting Information). Given that exchange of cholesterol with lipoproteins in serum could lead to cholesterol accumulation on RBC membranes, HCD diet may cause RBC to be more fragile and subsequently hemolysis. However, we did not observe changes in cell morphology, or osmotic fragility of red blood cells from HCD mice (Figure S7B,C, Supporting Information), suggesting the absence of a hemolytic anemia.

HCD mice showed normal body weight and a mildly enlarged spleen (Figure S7D,E, Supporting Information). The percentage of total TER119^+^ erythroid cells in bone marrow was significantly decreased in HCD fed mice compared with chow diet mice (Figure 7C). The defective erythropoiesis of bone marrow under the HCD condition is further characterized by the increase of nucleated erythroblasts and dramatically reduced reticulocytes and mature red blood cells in HCD mouse bone marrow, using CD44 and forward scatter to divide erythroid cells into different developmental stages (Figure 7D,E).^[^ ^49^ ^]^ To further confirm the defects in erythropoiesis in HCD mice, we induced stress erythropoiesis by phenylhydrazine (PHZ) administration.^[^ ^50^ ^]^ HCD mice exhibited more profound decreases in RBC, HCT, and Hb levels and subsequent delayed recovery after PHZ administration compared with control mice (Figure S7F, Supporting Information). TER119^+^ erythroid cells were also markedly decreased in HCD mouse bone marrow on day 9 post‐PHZ treatment, concomitant with increased nucleated erythroblasts and a decreased proportion of enucleated subsets (Figure S7G,H, Supporting Information). In addition, the percentage and cell number of hematopoietic stem and progenitor cells (HSPCs) were increased in bone marrow from HCD fed mice compared with that of chow diet mice, which is consistent with previous studies^[^ ^51^, ^52^ ^]^ (Figure S7I, Supporting Information). Common myeloid progenitors (CMPs) differentiated from HSPCs commit two distinct lineage biases: granulocyte‐macrophage progenitors (GMPs) and megakaryocyte‐erythrocyte progenitors (MEPs).^[^ ^53^ ^]^ The CMPs showed no change in bone marrow upon cholesterol diet feeding, whereas MEPs were increased with a compromise of GMPs in HCD mice compared with chow diet control (Figure S7J, Supporting Information). These data suggest that high cholesterol diet feeding impaired the normal hematopoiesis and induced lineage‐biased differentiation.

We also evaluated the effect of loss of LDLR on erythropoiesis which had been proved to elevate plasma cholesterol levels.^[^ ^54^, ^55^ ^]^ Indeed, the serum concentration of total cholesterol was significantly increased in LDLR KO mice compared with their WT littermates (Figure S8A,B, Supporting Information). Similar to that of high cholesterol diet feeding, loss of LDLR induced mild reduction of RBC and hemoglobin level, defective erythropoiesis in bone marrow, and mild splenomegaly (Figure S8C–G, Supporting Information). These data further supported that high cholesterol impaired the erythropoiesis in vivo.

### 2.8 Disruption of Cholesterol Synthesis Impairs Normal Erythropoiesis In Vivo

To assess the effect of cholesterol biosynthesis inhibition on the erythropoiesis in vivo, we pretreated mice with fatostatin and then challenged them with PHZ (Figure S9A, Supporting Information). These mice presented no obvious difference in red blood cell counts after 3‐days fatostatin treatment (day 0 in Figure S9B, Supporting Information). Interestingly, fatostatin treated mice exhibited a reduced capacity for erythropoiesis after PHZ administration (Figure S9B–D, Supporting Information). Moreover, fatostatin‐treated mice suffered from more serious splenic stress erythropoiesis (Figure S9E,F, Supporting Information). The percentage of nucleated erythroblasts in spleen from fatostatin‐treated mice was markedly increased due to stress erythropoiesis (Figure S9G, Supporting Information).

**Figure 8:**
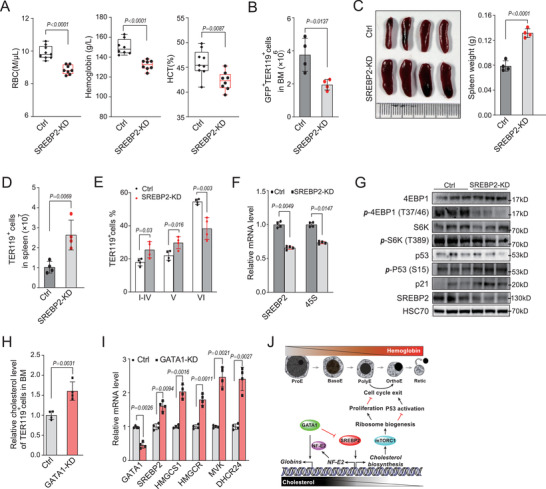
Disruption of cholesterol synthesis impairs the erythroblasts differentiation in vivo. A) Red blood cells (RBC), hemoglobin and hematocrit indices of indicated mice. Each dot represents one mouse. B) Quantification of percentage of GFP^+^ TER119^+^ cells in bone marrow from indicated mice (Control, *n* = 4; SREBP2‐KD, *n* = 4). C) Photomicrographs of spleens and statistical analysis of the spleen weight from indicated mice are shown in B). D) Quantification of percentage of TER119^+^ cells in spleen from indicated mice. E) Quantification of indicated populations in TER119^+^ erythroid cells from indicated mice spleen in D). F) Quantification of transcripts of pre‐rRNA 45S and SREBP2 in erythroblasts from indicated mice bone marrow in B). G) Western blot analysis of indicated proteins in erythroblasts from indicated mice bone marrow in B). HSC70 was used as a loading control. An equal number of cells were loaded in each well. H) Intracellular cholesterol levels were analyzed by flow cytometry based on the intensity of Filipin III staining of TER119^+^ cells from indicated mice. I) Quantification of transcripts of indicated genes in erythroblasts from indicated mice bone marrow. J) Schematic diagram of fine‐tuning of cholesterol homeostasis emerges as a key mechanism for regulating erythropoiesis. All *P* values were determined by unpaired two‐tailed Student's *t*‐test. See also Figures S9 and S10 (Supporting Information).

To further confirm the role of cholesterol biosynthesis in erythropoiesis, we performed bone marrow transplantation with lineage‐negative cells transduced with SREBP2 shRNA coexpressing GFP. SREBP2‐knockdown cell transplanted mice showed obvious anemia characterized by significantly lower RBC, hemoglobin level, and hematocrit and increase of immature reticulocytes in peripheral blood (**Figure** **8**A; and Figure S9H, Supporting Information). TER119^+^ erythroid cells derived from SREBP2 knockdown bone marrow was significantly decreased compared with control mice (Figure 8B; and Figure S9I,J, Supporting Information). Furthermore, these mice showed normal body weight but a significantly enlarged spleen (Figure 8C). The percentage of total TER119^+^ erythroid cells in spleen was significantly increased in SREBP2 knockdown mice, owing to increased nucleated erythroblasts and immature reticulocytes (Figure 8D,E).

Consistent with our in vitro findings, we found that SREBP2 knockdown mice and fatostain‐treated mice showed down‐regulation of 45S rRNA, decreased phosphorylation of 4EBP1 and S6K and increased phosphorylation of p53 and its transcriptional target p21 (Figure 8F,G; and Figure S9K,L, Supporting Information). However, reverse effects on these signaling were observed in HCD mice, including up‐regulation of 45S rRNA, mTORC1 activation, up‐regulation of ribosome biogenesis, and p53 inactivation (Figure 7F,G). These data demonstrate that disruption of cholesterol homeostasis impairs erythropoiesis in vivo via the cholesterol/mTORC1/ribosome biogenesis axis and p53 activation.

### 2.9 GATA1 Inactivation Leads to Defect of Erythropoiesis but Enhanced SREBP2 Activity In Vivo

To further confirm the role of GATA1 in the regulation of SREBP2 in vivo, we performed bone marrow transplantation with lineage‐negative cells transduced with GATA1 shRNA coexpressing GFP. GATA1‐knockdown cell transplanted mice showed significantly reduced RBC, hemoglobin level, and hematocrit (Figure S10A, Supporting Information). TER119^+^ erythroid cells derived from GATA1 knockdown bone marrow was significantly decreased compared with control mice (Figure S10B–D, Supporting Information). Furthermore, these mice showed normal body weight but a mildly enlarged spleen (Figure S10E, Supporting Information). The percentage of total TER119^+^ erythroid cells in spleen was significantly increased in GATA1 knockdown mice, owing to increased immature reticulocytes (Figure S10F,G, Supporting Information). Consistent with our in vitro findings, we found that GATA1 knockdown mice showed up‐regulation of SREBP2 and cholesterol biosynthetic genes (Figure 8H), and increase of cholesterol levels of TER119^+^ cells from both bone marrow (Figure 8I) and spleen (Figure S10H, Supporting Information).

## 3 Discussion

In this study, we have uncovered a critical role for cholesterol homeostasis in terminal erythropoiesis: disrupting intracellular cholesterol levels interfered with terminal erythroid differentiation and resulted in anemia. On the other hand, we have shown that cholesterol biosynthesis is downregulated in the late stages of terminal erythroid differentiation, leading to the gradual reduction in intracellular cholesterol levels (Figure 4). This is consistent with the widespread downregulation of gene expression, with the exception of red blood cell‐related genes.^[^ ^56^ ^]^ Reduced cholesterol in later stages functions as a brake for cell proliferation, to promote cell cycle exit that is crucial for enucleation (Figure 2). Moreover, cholesterol biosynthesis is an energetically expensive process requiring significant inputs from acetyl‐CoA, ATP, oxygen, and the reducing factors Nicotinamide adenine dinucleotide phosphate (NADPH) and nicotinamide adenine dinucleotide (NADH).^[^ ^1^ ^]^ Downregulation of cholesterol biosynthesis provides an energy‐saving strategy for the major actions of terminal erythroid maturation including enucleation. Together, our work demonstrates that fine‐tuning of cholesterol homeostasis emerges as a key mechanism for erythropoiesis regulation (Figure 8J).

Ribosome biogenesis is an essential cellular process, and its regulatory role in erythropoiesis has been implied by the pathological states in macrocytic anemia, such as Diamond–Blackfan anemia or acquired 5q‐ syndrome that are caused by point mutations or deletions in ribosomal proteins.^[^ ^40^ ^]^ It has been documented that ribosome levels regulate translation of select transcripts and erythroid lineage commitment.^[^ ^57^ ^]^ Furthermore, ribosome biogenesis is downregulated when cells enter the late stages of erythroid maturation in human and mouse models, where erythroblasts are switched from proliferation to differentiation.^[^ ^58^, ^59^ ^]^ Notably, our study revealed a dynamic change in ribosome biogenesis that is increased at the early stages, followed by a decrease toward later stage of erythropoiesis. Consistent with our findings, a recent study using an in vitro human erythroblast culture system also reported that ribosome biogenesis is abruptly interrupted by a drop in rDNA transcription and collapse of ribosomal protein neo‐synthesis during terminal erythropoiesis, coinciding with p53 activation.^[^ ^60^ ^]^ Premature inhibition of ribosome biogenesis induced by RNA polI inhibitor CX‐5461 resulted in p53 activation that drove cell cycle arrest of erythroblasts, suggesting that ribosome biogenesis downregulation may function as a brake for cell proliferation to promote cell cycle exit during normal erythroid differentiation. In line with this, increase of 45S rRNA transcription characterized by the elevated 45S rRNA transcription, concomitant with inhibition of p53 activation and p21 expression, led to enhanced cell proliferation and continuous cell cycling induced by excess cholesterol, which is markedly restored by CX‐5461 (Figure 3). Taken together, our data provides strong evidence that the timing of ribosome biogenesis extinction and p53 activation are crucial for normal terminal erythroid differentiation.

mTORC1 has been shown to play distinct roles at different stages of erythropoiesis, and its perturbations result in anemia.^[^ ^61^, ^62^ ^]^ The requirements of mTORC1 activation for erythroid progenitor proliferation and mTORC1 inhibition for terminal enucleation are highly relevant with our findings. It is possible that high levels of cholesterol activate mTORC1 and subsequent ribosome biogenesis to enhance protein and lipid synthesis that are required for the rapid cell divisions in the early stage of terminal differentiation, while lower cholesterol inhibits mTORC1 activity and ribosome biogenesis and activates p53, thereby coordinating termination of cell proliferation and cell cycling in the later stage of erythroid differentiation. Our study revealed the role of the cholesterol/mTORC1/ribosome biogenesis axis in control of cell proliferation and cycling in erythropoiesis, providing new insights into the functions of lipid metabolism in erythropoiesis. We did not exclude the possibility that intermediate products (and not cholesterol) from the cholesterol synthesis pathway regulates erythroid differentiation during primitive erythropoiesis. Indeed, isoprenoids have been reported to regulate erythropoiesis by modulating the expression of the GATA1 transcription factor.^[^ ^22^ ^]^

We also show that GATA1 binds SREBP2 to downregulate cholesterol biosynthesis that is required for the terminal erythropoiesis. This is consistent with the increased GATA1 expression as cell differentiates from the CFU‐E to orthochromatic erythroblast stages.^[^ ^63^ ^]^ Thus, GATA1 per se may actively regulate the cell cycle exit and reduction of cell size through inhibition of SREBP2‐mediated cholesterol biosynthesis. Since SREBP1 and SREBP2 are 47% identical, it will be interesting to determine whether GATA1 can also bind and inhibit SREBP1 activity to reduce fatty acid synthesis and thus phospholipid level of cell membranes.^[^ ^22^ ^]^ Furthermore, our data identifies that SREBP2 directly regulates NFE2 expression by binding to its promoter. Given the importance of maintaining the balance of globin and heme during erythroid differentiation,^[^ ^64^, ^65^ ^]^ a GATA1/SREBP2/NFE2 axis provides a feedback loop to regulate the globin expression, which protects late stage erythroblasts from overproduced globin.

GATA family of transcription factors play complex and widespread roles in cell fate decisions and tissue morphogenesis.^[^ ^66^ ^]^ GATA factors binding GATA sequence motifs via a highly conserved dual zinc‐finger domain, are essential for the development of tissues derived from all three germ layers, including the skin, brain, gonads, liver, hematopoietic, cardiovascular, and urogenital systems.^[^ ^67^, ^68^ ^]^ Similar regulatory mechanisms cholesterol homeostasis via GATAs binding SREBP2 could also occur in these cell types. Our study may shed light on the possible mechanisms by which cells switch from proliferation to differentiation in these developmental systems.^[^ ^69^ ^]^

In summary, our study has identified a critical role for cholesterol homeostasis during erythroblast differentiation, which may provide clues for the pathogenesis of erythroid related diseases of unknown etiology. This is especially relevant in patients with high‐erythropoietic activity anemia and myelodysplastic syndromes in which a failure of cell cycle exit is one of the key features of dyserythropoiesis.

## 4 Experimental Section

### Mice and Treatment

C57BL/6 and LDLR knockout mice were purchased from Charles River Laboratories (Bei Jing, China). Mice were maintained on standard rodent diet. For the high cholesterol diet feeding, the mice were switched to regular AIN‐93G rodent chow diet or AIN‐93G diet containing 2.5% purified cholesterol (Trophic, Nanjing, China). After 4 weeks, the mice were used for indicated experiments. To assess the effect of inhibition of cholesterol synthesis on the mice under PHZ‐induced stress erythropoiesis, mice were pretreated with vehicle Dimethyl sulfoxide (DMSO) or fatostatin (30 mg kg^−1^) for 3 days before PHZ injection on day 0. Mice were then subjected to daily DMSO or fatostatin injections during day 0–6. Peripheral blood was collected from the tail vein into ethylene diamine tetraacetic acid (EDTA)‐treated tubes and analyzed using a BC‐5150 (Mindray, Shenzhen, China) at different time points. All drugs were administrated by intraperitoneal injection. Both males and females at 8–10 weeks of age were included in each group.

For transplantation of SREBP2‐knockdown or GATA1‐knockdown cells, lineage negative bone marrow cells were isolated and infected with virus encoding SREBP2, GATA1, or Scramble shRNA, then transplanted into irradiated recipient mice (8–10 weeks old) by retro‐orbital injection.

All animal studies were performed in accordance with the Guidelines for the Care and Use of Laboratory Animals and were approved by the Institutional Animal Care and Use Committees at Shandong University (#19 026). Animals were euthanized using CO_2_.

### Plasmids Construction and Cell Transduction

Retroviral shRNA oligonucleotides against SREBP2, GATA1, and NFE2 were provided in Table S7 in the Supporting Information. Cloning of shRNA into MSCV‐U3‐H1 retroviral vector was performed as previously described.^[^ ^70^ ^]^ The gene overexpression plasmids were constructed by standard molecular cloning: MSCV‐SREBP2 encodes active SREBP2(1‐468aa), pLENTI‐GFP‐GATA1 encodes human GATA1 with indicated domain (FL: 1‐1239 aa; NTD: 1‐594aa; ZF: 595‐954aa; CTD: 954‐1239aa) with a GFP epitope tag, pLENTI‐FLAG‐SREBP2 encodes active human SREBP2(1‐468aa) with a FLAG tag, and pLENTI‐FLAG‐SREBP2‐FL encodes full length of human SREBP2 (1‐3387aa) with 3× FLAG tag. Generation of retroviral particles and viral infection of cells were performed as previously described.^[^ ^16^ ^]^

### Cell Culture

K562, HEK293, and HeLa cells were purchased from the Cell Bank of the Shanghai Institute for Biological Sciences, Chinese Academy of Science. G1E‐ER4 was kindly provided by Prof. Xiang Lv and Prof. Mitchell J Weiss. HEK293 and HeLa cells were cultured in Dulbecco's modified eagle medium (DMEM) medium (Gibco), and K562 cells were cultured in RPMI 1640 medium (Gibco) supplemented with 10% fetal bovine serum (Biological Industries); G1E‐ER4 cells were cultured in Iscove's Modified Dulbecco's Medium (IMDM) medium (Gibco) containing 15% fetal bovine serum (FBS) (Biological Industries), 45 × 10^−6^ m monothioglycerol (Sigma), 100 ng mL^−1^ SCF (Pepro Tech) and 3 U mL^−1^ recombinant human Epo (Amgen). Lineage negative bone marrow cells were cultured in StemSpan medium (StemCell Technologies) supplemented with 10 ng mL^−1^ mouse IL‐3 (Peprotech), 10 ng mL^−1^ IL‐6 (Peprotech), 50 ng mL^−1^ mouse stem cell factor (Peprotech), and 20 µg mL^−1^ human low‐density lipoprotein (StemCell Technologies).

Purification of mouse fetal liver TER119‐negative cells (FLCs) and in vitro culture were performed as previously described.^[^ ^30^, ^31^, ^71^ ^]^ In brief, total fetal liver cells were labeled with biotin‐conjugated TER119 antibody and were purified through a StemSep column. Purified TER119 negative cells were cultured in Epo containing medium for 24 or 48 h to analyze erythroid differentiation. As for virus transduction, purified TER119 negative cells were subsequently cultured in medium containing growth factors other than erythropoietin (Epo) to maintain their progenitor stage for 12 h allowing the exogenous cDNAs or shRNAs to express, and cells were then changed to Epo medium for culture 24 or 48 h to induce cell differentiation.

### Coimmunoprecipitation

The cells were harvested and suspended in 700 µL Pierce IP lysis buffer (Thermo Fisher Scientific) supplemented with a proteinase inhibitor cocktail and incubated on ice for 30 min followed by centrifugation at 13 000 rpm for 15 min at 4 °C. The cleared cell lysates were immunoprecipitated with beads conjugated with indicated antibodies at 4 °C for 12 h. The beads were then washed with IP buffer five times and boiled at 95 °C in 2× NuPAGE LDS Sample Buffer (Thermo Fisher Scientific) for 5 min. Aliquots were analyzed by immunoblotting.

### RNA‐seq and GSEA Analysis

TER119‐negative fetal liver erythroblasts were purified and cultured in Epo medium for 30 h supplemented with or without cholesterol (40 × 10^−6^ m), then cells were immunostained with TER119 antibody and positive cells were sorted by flow cytometer (Beckman). G1E‐ER4 cells were transduced with retrovirus to express FLAG tagged, active SREBP2 (1‐468aa) (FLAG‐nSREBP2), cells were then treated with Vehicle or *β*‐estradiol (500 × 10^−9^ m) for 6 h. Total RNA was extracted using the TRIzol reagent according to the manufacturer's instructions (Invitrogen). RNA quality and quantity were determined using a Nano Drop and Agilent 2100 bioanalyzer (Thermo Fisher Scientific). RNA sequencing was conducted using the BGIseq500 platform (Shenzhen, China). The GSEA was performed using the RNA‐sequencing gene expression profiling data and GSEA software and the Molecular Signatures Database (Broad Institute, USA). RNA sequencing data that support the findings of this study were deposited in NCBI SRA under accession number GSE166153 and GSE184002.

### Flow Cytometric Assays

Single‐cell suspensions of bone marrow, spleen, or cultured fetal liver cells were prepared and stained as previously described.^[^ ^72^ ^]^ Detailed information of the antibodies used in this study is shown in Table S6 in the Supporting Information.

### Fluorescence In Situ Hybridization (FISH)

FISH was performed with a FISH Testing Regent Kit (Abiocenter, Inc, Wuxi, China) according to the manufacturer's protocol. In brief, cells were fixed with 4% paraformaldehyde and permeabilized in 95% methanol, and hybridization was performed at 37 °C for 12 h in provided buffer with 5’ETS probe conjugated to Alexa 647 (5’AGACGAGAACGCCTGACACGCACGGCAC3’). Images were obtained using an Axio Observer A1 confocal fluorescence microscope (ZEISS, Germany).

### ChIP Analysis

ChIP assay was performed with a SimpleChIP Enzymatic Chromatin IP Kit (Cell Signaling Technology, Danvers, MA) according to the manufacturer's protocol. In brief, G1E‐ER4 cells were transduced with retrovirus to express FLAG‐nSREBP2 (1‐468aa), cells were then treated with Vehicle or *β*‐estradiol (200 × 10^−9^ m) for 3 h, fixed with 1% formaldehyde, and immuno‐precipitated with anti‐IgG or anti‐FLAG (Sigma) beads, the amount of bound DNA was quantified by quantitative PCR with different sets of primers as indicated. The primers were provided in Table S7 in the Supporting Information.

### Luciferase Reporter Assay

To evaluate the transcriptional activity of SREBP2 on the NFE2 promoter, 400 ng of the promoter plasmid pGL3‐NFE2 was co‐transfected with either expression plasmids FLAG‐SREBP2 (1‐468aa) or an empty vector. 40 ng of the pRL‐MCV plasmid (Renilla luciferase) (Promega) was included as an internal control. Plasmids were transfected with 3 µL of Lipofectamine 2000 (Thermo Fisher Scientific) into HEK293 cells in a 12‐well cell culture plate for 24 h. Dual‐Luciferase Reporter Assay System (Promega, Madison, WI) was used to determine the transcriptional activity of the promoter. The fold change of relative luciferase intensity was calculated based on the ratio of the Firefly and Renilla luciferase activities.

### Statistical Analysis

All statistical analyses were performed using Prism 8 (GraphPad Software). All data are presented as mean ± SD except where indicated otherwise. All comparisons were tested using unpaired two‐tailed Student's *t*‐test or one‐way ANOVA as described in the figure legends. A *P* value of less than 0.05 was considered statistically significant. Blinding was performed for in vitro experiments with data analysis by different operators.

Detailed information of the antibodies, reagents, and primer sequences used in this study is provided in Tables S6 and S7 (Supporting Information).

## Conflict of Interest

The authors declare no conflict of interest.

## Author Contributions

Z.L. and L.H. contributed equally to this work. Conceptualization, J.L. and B.Z.; Investigation, Z.L., L.H., Y.L., Y.X., R.Z., Q.Z., Q.S., Y.L., and J.C.; Writing–Original Draft, Z.L., L.H., J.L., and B.Z.; Writing–Review & Editing, Y.S., J.L., and B.Z.; Funding Acquisition, Y.S., J.L. and B.Z.; Supervision, J.L. and B.Z.

## Supporting information

Supporting Information

Supplemental Figure 1

Supplemental Table 1

Supplemental Table 2

Supplemental Table 3

Supplemental Table 4

Supplemental Table 5

## Data Availability

The Data that support the findings of this study are available in the Supporting Information of this article. The RNA‐seq data that support the findings of this study will be openly available in Gene Expression Omnibus, GEO at https://urldefense.com/v3/__https://www.ncbi.nlm.nih.gov/geo/__;!!N11eV2iwtfs!‐YoQAm1XgFFDNScFsk0UKsOWUrqUApQN3a7HUbQwlhMkCGbSjlpnQ1dndYWmb90d6C4$ , reference number GSE166153 and GSE184002.
