# Supplementary material for: Fine‐Tuning of Cholesterol Homeostasis Controls Erythroid Differentiation
Source: Adv Sci (Weinh). 2021 Nov 5;9(2):2102669. doi: 10.1002/advs.202102669 (PMC8805577; doi:10.1002/advs.202102669)
Supplement: Supplementary file 1 — Supporting Information [file ADVS-9-2102669-s001.pdf]

ADVANCED SCIENCE
Open Access

Supporting Information

for *Adv. Sci.*, DOI: 10.1002/advs.202102669

Fine-tuning of Cholesterol Homeostasis Controls Erythroid Differentiation

Zhiyuan Lu, Lixia Huang, Yanxia Li, Yan Xu, Ruihao Zhang, Qian Zhou, Qi Sun, Yi Lu, Junjie Chen, Yuemao Shen, Jian Li, and Baobing Zhao**

Supplemental Information

Fine-tuning of cholesterol homeostasis controls erythroid differentiation

Zhiyuan Lu, Lixia Huang, Yanxia Li, Yan Xu, Ruihao Zhang, Qian Zhou, Qi Sun, Yi Lu, Junjie Chen, Yuemao Shen, Jian Li*, Baobing Zhao**

Supplemental Figure legends

Figure S1 Inhibition of cholesterol biosynthesis affects normal terminal erythroid differentiation, related to Figure 1.

(**A**) Quantification of percentage (left) and cell number of TER119$^+$ cells (right) in differentiated erythroblasts after treatment with U18666A for 24 h. FLCs were purified and cultured in Epo containing medium. (**B**) Statistical analysis of the percentage of reticulocytes after treatment with U18666A for 48 h. FLCs were purified and cultured in Epo containing medium. (**C**) Quantification of percentage (left) and cell number of TER119$^+$ cells (right) in differentiated erythroblasts after treatment with fatostatin for 24 h. (**D**) Statistical analysis of reticulocytes after FLCs were treated with fatostatin for 48 h. (**E**) Quantification of percentage (left) and cell number of TER119$^+$ cells (right) in differentiated erythroblasts after treatment with indicated compounds for 24 h. FLCs were cultured as in (A). CH indicated cholesterol. (**F**) FLCs were cultured with or without indicated dose of lovastatin for 24 h, and percentage (left) and number of TER119$^+$ cell (right) was measured by flow cytometry. (**G-H**) FLCs were cultured in Epo containing medium and treated with YM53601. Percentage and cell number of TER119$^+$ cells (G) were measured by flow cytometry at 24 h. Percentage of reticulocytes (H) was measured by flow cytometry at 48 h using TER119 and Hoechst staining. (**I-P**) FLCs were cultured in Epo containing medium for 30 h, then cells were treated with fatostatin (I-G), lovastatin (K-L), Lipoprotein deficient serum (LPDS, M-N), or U18666A (O-P) for additional 18 h, cell number of TER119$^+$ cell and percentage of reticulocytes were analyzed by flow cytometry. (**Q**) Quantification of the mRNA expression of *SREBP2* in cells from Figure 1G. *P* values were determined by using unpaired two-tailed Student's t-test (G, H) or 1-way ANOVA with Tukey's multiple comparisons test (A-F, Q). Data are presented as mean ± SD from three independent

experiments.

Figure S2 Excess cholesterol disrupts normal cell cycle process and erythroid differentiation in the late stages of terminal erythropoiesis, related to Figure 2.

(**A**) Morphologic analysis of erythroid differentiation after *in vitro* culture for 48 h by benzidine-Giemsa staining. Ctrl: control; CH: cholesterol. Arrows indicated reticulocytes. (**B**) FLCs were cultured in Epo medium for 30 h, and then treated with or without cholesterol (40 μM) for additional 18 h. Cell number of $TER119^{+}$ cells and percentage of reticulocytes were analyzed by flow cytometry using TER119 and Hoechst staining. (**C**) Quantification of the mRNA expression of human *SREBP2* in cells from Figure 2E. (**D**) Representative cell cycle profiles of erythroblasts after 30 h of *in vitro* culture with or without cholesterol (40 μM). Cell cycle was analyzed by flow cytometry using propidium iodide staining. Percentage of cells in indicated phases is shown. (**E**) Quantification of cells in indicated phases from (D). (**F**) Representative cell cycle profiles of erythroblasts after 30 h of *in vitro* culture with normal FBS (Ctrl) or lipoprotein deficient serum (LPDS). (**G**) Quantification of cells in indicated phases from (F). All *P* values were determined by unpaired two-tailed Student's t-test. Data are presented as mean ± SD from three independent experiments.

Figure S3 Ribosome biogenesis is downregulated during terminal erythropoiesis, related to Figure 3.

(**A**) Gene Set Enrichment Analysis of sterol biosynthetic pathway in erythroblasts treated by cholesterol (CH, 40 μM) or vehicle (control, Ctrl). Cell was treated as in Figure 3A. (**B**) Quantification of pre-rRNA 45S transcripts in E14.5 mouse fetal liver cells shown as in Figure 4D. (**C**) Quantification of pre-rRNA 45S transcripts in cultured erythroblasts. D0 to D2 indicate different days of TER119 negative mouse fetal liver erythroblasts *in vitro* culture. (**D**) Polysome profiling from cells treated as in Figure 3C. (**E**) Gene Set Enrichment Analysis of cell cycle pathway in erythroblasts treated by cholesterol (CH, 40 μM) or vehicle (control, Ctrl). Cell was treated as in Figure 3A. (**F**) Quantification of relative protein levels of Figure 3I. (**G**) Quantification of relative protein levels of Figure 3J. (**H**) Western blot analysis of indicated proteins in erythroblasts after 30 h of *in vitro* culture with lipoprotein deficient serum (LPDS) or plus with cholesterol (CH, 40 μM). HSC70 was used as a loading control. An equal number of cells were loaded in each well. All *P* values were determined by unpaired two-tailed

Student's t-test. Data are presented as mean ± SD from three independent experiments.

Figure S4 Cholesterol synthesis is down-regulated during terminal erythroid differentiation, related to Figure 4.

(**A**) Quantification of the mRNA level of indicated genes in K562 cells after incubation of hemin for 24 h. (**B**) Schematic diagram of cholesterol biosynthesis pathway. Cholesterologenic enzymes and relative inhibitors are highlighted in green and red respectively. (**C**) Quantification of the mRNA level of indicated genes in Hela cells after incubation of hemin (40 μM) for 24 h. (**D**) Quantification of the mRNA level of genes encoding cholesterologenic enzymes in K562 cells after incubation of butyrate (1 mM) for 48 h. (**E**) Representative flow cytometric profiles of cellular phosphatidylserine in indicated cells as in Figure 4D using annexin V staining. (**F**) Representative flow cytometric profiles of erythroid populations at different developmental stages in bone marrow. Cells were gated based on cell size and CD44 expression. Populations I to VI represent the least differentiated to enucleated RBC. (**G**) Quantification of the mRNA expression of indicated genes in cells sorted according to (F). Transcripts were normalized to 18S ribosomal RNA. Data are presented as mean ± SD from three independent experiments.

Figure S5 GATA1 downregulates cholesterol biosynthesis, related to Figure 5.

(**A**) Quantification of indicated genes in G1-ER4 cells after induction of β-estradiol (EST, 500 nM). Data are presented as mean ± SD from three independent experiments. (**B**) Quantification of indicated genes in G1-ER4 cells with GATA1 knockdown. shGATA1-2 represented a different shRNA targeting GATA1 with the one used in Figure 5B. (**C**) K562 cells were transduced with lentivirus encoding GATA1 shRNA and the mRNA expression of *GATA1* and cholesterol synthesis related genes were analyzed by quantitative PCR. (**D**) Immunoblot analysis of SREBP2 activation in K562 cells. K562 cells stably expressing FLAG-nSREBP2 were cultured with normal FBS or lipoprotein deficient serum (LPDS) supplemented with 5 μM lovastatin, and then incubated with 40 μM hemin or vehicle for indicated time. (**E**) Quantification of the mRNA expression of human SREBP2 in cells from Figure 5C. (**F**) Co-IP of endogenous SREBP2 and GATA1 in K562 cells with or without incubation of hemin. (**G**) Structure of full-length and active SREBP2. TM: transmembrane; bHLH-Zip: basic helix-loop-helix; C-reg: c-regulatory. Transcripts were normalized to 18S ribosomal RNA. *P* value was determined by unpaired two-tailed Student's t-test. Data are presented as mean ± SD from three

independent experiments.

Figure S6 Transcriptional control of NFE2 by SREBP2 contributes to the regulation of globin expression GATA1 downregulates cholesterol biosynthesis, related to Figure 6.

(**A**) A heatmap of representative GATA1 target genes with or without EST stimulation (n=3, adjusted p value [p-adj] < 0.05). (**B**) A heatmap of the genes encoding cholesterologenic enzymes upregulated by SREBP2 overexpression (n=3, adjusted p value [p-adj] < 0.05). (**C**) Venn diagram indicated the number of differential genes and common genes between SREBP2 overexpression and EST treatment (1.2-fold change cutoff). (**D**) Immunoblot analysis of nuclear localization of the GATA1/ER proteins induced by SREBP2 overexpression or EST. GAPDH and Lamin B1 were the endogenous markers for cytosolic and nuclear proteins. (**E**) Quantification of the mRNA expression of human *SREBP2* in cells from Figure 6C. Transcripts were normalized to 18S ribosomal RNA. *P* value was determined by unpaired two-tailed Student's t-test. Data are presented as mean ± SD from three independent experiments.

Figure S7 Excess cholesterol impairs erythropoiesis *in vivo*, related to Figure 7.

(**A**) White blood cells (WBC) and platelets indices of indicated mice. C57BL/6J mice were fed on chow diets (n=9) or high cholesterol diets (HCD) (n=9) for 4 weeks. Both males and females were included in each group. (**B**) Representative morphologic analysis of peripheral blood smear from (A) by benzidine-Giemsa staining. (**C**) The osmotic fragility and hemolysis of red blood cells from indicated mice from (A). (**D-E**) Statistical analysis of body and spleen weight of indicated mice from (A). (**F**) Indicated mice from (A) were injected with PHZ on day 0. Red blood cells (RBC), hemoglobin and hematocrit indices were measured on indicated days after PHZ injection. Data are presented as mean ± SD from 9 mice for each group. $^{**}P < 0.01$, $^{***}P < 0.001$ and $^{****}P < 0.0001$. (**G**) Quantification of TER119^{+} cells in bone marrow from indicated mice on day 9 after PHZ injection. Each dot represents one mouse. (**H**) Quantification of indicated populations from (G). Populations I to VI represent the least differentiated to enucleated RBC as shown as in Figure 7D. Data are presented as mean ± SD from 4 mice for each group. $^{****}P < 0.0001$. (**I**) Quantification of percentage and cell number of hematopoietic stem and progenitor cells (HSPCs) in HCD mice bone marrow from Figure 7E. Each dot represents one mouse. (**J**) Quantification of indicated populations in HSPC cells from indicated mice bone marrow from (H). HSPCs are divided into three subpopulations which are common

myeloid progenitors (CMPs), granulocyte-macrophage progenitors (GMPs) and megakaryocyte-erythrocyte progenitors (MEPs) according to CD16/32 and CD34. Each dot represents one mouse. Data are presented as mean ± SD from 4 mice for each group. *P* values were determined by using unpaired two-tailed Student's t-test (A, E, G, I, G, H) or 1-way ANOVA with Tukey's multiple comparisons test (F).

Figure S8 Loss of LDLR impairs erythropoiesis *in vivo*, related to Figure 7.

(**A**) Western blot analysis of LDLR proteins in erythroblasts from indicated mice bone marrow. α-tubulin was used as a loading control. An equal number of cells were loaded in each well. (**B**) Quantification of serum cholesterol in LDLR knockout mice. Each dot represents one mouse. Five males at the age of 8 weeks were included in each group. (**C**) Red blood cells (RBC), hemoglobin and hematocrit indices of indicated mice from (B). (**D**) Quantification of percentage of $TER119^+$ cells in bone marrow of indicated mice from (B). (**E**) Quantification of indicated populations in $TER119^+$ cells from (D). Populations I to VI represent the least differentiated to enucleated RBC. (**F**) Photomicrographs of spleens and statistical analysis of the spleen weight from indicated mice are shown in (B). (**G**) Quantification of indicated populations in $TER119^+$ cells from indicated mice spleen in (F). All *P* values were determined by unpaired two-tailed Student's t-test. Data are presented as mean ± SD from 5 mice for each group.

Figure S9 Disruption of cholesterol synthesis impairs normal erythropoiesis *in vivo*, related to Figure 8.

(**A**) Experimental design for fatostatin-treated mice in response to PHZ-induced hemolytic anemia. Red arrows indicate the timepoint for complete blood count test. All drugs were administrated by intraperitoneal injection. Both males and females were included in each group. (**B**) Red blood cells (RBC), hemoglobin and hematocrit indices of indicated mice in (A). Each dot represents one mouse. Data are presented as mean ± SD from 6 mice for each group. (**C**) Quantification of $TER119^+$ cells in bone marrow from indicated mice on day 9 after PHZ injection. Data are presented as mean ± SD from 4 mice for each group. (**D**) Quantification of indicated populations in $TER119^+$ erythroid cells from indicated mice bone marrow in (C). Populations I to VI represent the least differentiated to enucleated RBC as shown as in Figure 7D. Data are presented as mean ± SD from 4 mice for each group. $**P < 0.01$, $***P < 0.001$

and ****P < 0.0001. (**E**) Photomicrographs of spleens from indicated mice as in (C). (**F**) Statistical analysis of the spleen-to-body-weight ratio (Vehicle, n=4; fatostatin, n=4). (**G**) Quantification of indicated populations in TER119^{+} erythroid cells from indicated mice spleen in (C). (**H**) Representative morphologic analysis of peripheral blood smear from indicated mice as in Figure 8A by benzidine-Giemsa staining. (**I**) Quantification of percentage of GFP^{+} cells in indicated groups before and in bone marrow cells after 4 weeks transplantation respectively. (**J**) Quantification of percentage of GFP^{+}TER119^{+} cells in bone marrow from indicated mice at 4 weeks after transplantation. Data are presented as mean ± SD from 4 mice for each group. (**K**) Quantification of transcripts of pre-rRNA 45S in erythroblasts from indicated mice bone marrow as in (C). (**L**) Western blot analysis of indicated proteins in erythroblasts from indicated mice bone marrow as in (C). HSC70 was used as a loading control. An equal number of cells were loaded in each well. All P values were determined by unpaired two-tailed Student's t-test.

Figure S10 Knockdown GATA1 promotes cholesterol synthesis *in vivo*, related to Figure 8.

(**A**) Red blood cells (RBC), hemoglobin and hematocrit indices of indicated mice at 4 weeks after transplantation (Control, n=4; GATA1-KD, n=4). Each dot represents one mouse. (**B**) Quantification of cell number of GFP^{+}TER119^{+} cells in bone marrow from indicated mice in (A). (**C**) Quantification of indicated populations in TER119^{+} cells from indicated mice bone marrow in (B). (**D**) Quantification of percentage of GFP^{+} cells in indicated groups before and in bone marrow cells after 4 weeks transplantation respectively. (**E**) Photomicrographs of spleens and statistical analysis of the spleen weight from indicated mice are shown in (B). (**F**) Quantification of percentage of TER119^{+} cells in spleen from indicated mice. (**G**) Quantification of indicated populations in TER119^{+} erythroid cells from indicated mice spleen in (F). (**H**) Intracellular cholesterol levels were analyzed by flow cytometry based on the intensity of Filipin III staining of TER119^{+} cells from indicated mice in (B). *P < 0.05, **P < 0.01 and ***P < 0.001. All P values were determined by unpaired two-tailed Student's t-test. Data are presented as mean ± SD from 4 mice for each group.

Supplemental Tables

Table S1 Gene Set Enrichment Analysis of genes downregulated in erythroblasts treated by cholesterol (*p* value< 0.01; FDR *q* value <0.01). TER119-negative fetal liver erythroblasts were purified and cultured in Epo medium for 30 h supplemented with or without cholesterol (40 μM), and then TER119-positive cells were sorted by flow cytometry and subjected to RNA deep sequencing. GSEA of the transcriptome to reveal enriched GO_BP terms. Related to the file of Table S1.

Table S2 Gene Set Enrichment Analysis of genes upregulated in erythroblasts treated by cholesterol (*p* value< 0.01; FDR *q* value <0.01). The cells were treated as in Table S1. GSEA of the transcriptome to reveal enriched GO_BP terms. Related to the file of Table S2.

Table S3 DAVID analysis of overlapped genes in G1E-ER4 cells shared by SREBP2 overexpression and EST treatment respectively. (*p* value< 0.05; FDR <0.25). G1E-ER4 cells transduced with retrovirus to express active SREBP2 (1-468aa) or blank vector control, were treated with Vehicle or β-estradiol (500 nM) for 6 h respectively, and then subjected to RNA deep sequencing. DAVID analysis was performed with genes oppositely regulated in both groups (1-fold change cutoff). Related to the file of Table S3.

Table S4 Gene Set Enrichment Analysis of genes upregulated in G1E-ER4 cells with SREBP2 overexpression and EST stimulation. (*p* value< 0.05; FDR <0.25). G1E-ER4 cells were transduced with retrovirus to express active SREBP2 or blank vector control, and then treated with β-estradiol (500 nM) for 6 h and subjected to RNA deep sequencing. GSEA of the transcriptome to reveal enriched GO_BP terms and KEGG pathways. Related to the file of Table S4.

Table S5 Gene Set Enrichment Analysis of genes downregulated in G1E-ER4 cells with EST stimulation. (*p* value< 0.05; FDR <0.25). G1E-ER4 cells transduced with retrovirus to express blank vector control, were treated with Vehicle or β-estradiol (500 nM) for 6 h and subjected to RNA deep sequencing. GSEA of the transcriptome to reveal enriched GO_BP terms and KEGG pathways. Related to the file of Table S5.

Table S6. Antibodies and commercial reagents used in this study.

**Antibodies/Regents**	**Source**	**Catalog Number**
LDLR antibody	ABclonal	A14996
GATA1 antibody	Cell Signaling Technology (CST)	D52H6
α-Tublin antibody	Proteintech	11224-1-AP
GFP antibody	Proteintech	66002-1-lg
SREBP2 antibody	Proteintech	28212-1-AP
HSP70 antibody	Proteintech	10995-1-AP
Lamin B1	Proteintech	12987-1-AP
NF-E2 antibody	Proteintech	11089-1-AP
FLAG-tag antibody	Sigma	F3165
RPS19 antibody	Proteintech	15085-1-AP
P53 antibody	abcam	ab26
p-P53 antibody	abcam	Ab1431
P21 antibody	abcam	ab109520
4EBP1 antibody	CST	9644
p-4EBP1 antibody	CST	2855
S6K antibody	Proteintech	14485-1-AP
p-S6K antibody	CST	9205
Biotin Mouse Lineage Panel	BD Bioscience	559971
Biotin-TER119	eBioscience	13-5921-85
APC-TER119 antibody	eBioscience	47-5921-80
PE-CD44 antibody	eBioscience	12-0441-81
FITC-Ki-67 antibody	eBioscience	11-5698-82
PE-CD71 antibody	eBioscience	11-0711-82
PE/CY7-CD34	Biolegend	119326
PB-Lineage antibody	Biolegend	133306
PE-CD16/32	Biolegend	101308
FITC-Sca-1	eBioscience	11-5981-85
APC-CD117	eBioscience	17-1171-83

hemin	Sigma	51280
cholesterol	Sigma	C3045
Methyl-β-cyclodextrin	Sigma	C4555
Hexadimethrine bromide	Sigma	H9268
Lovastatin	Selleckchem	S7645
Sodium butyrate	Sangon Biotech	156-54-7
Filipin complex	Sigma	SAE0088
β-Estradiol	Sigma	E2758
Fatostatin	MCE	125B11
YM53601	MCE	HY-100313
Amplex® Red Cholesterol Assay Kit	Invitrogen	A12216
Dual-Luciferase® Reporter Assay System	Promega	REF.E1910
PrimerScript RT reagent	Takara	RR047A
SYBR Premix Ex Taq	Takara	RR420A
The SimpleChIP® Enzymatic Chromatin IP Kit (Magnetic Beads)	CST	9003
FISH Testing Regent Kit	Abiocenter	AF2010SG010

Table S7. Primer sequences used in this study.

Genes	Oligonucleotides
Human SQLE primer	Forward：5’-GATGATGCAGCTATTTTCGAGGC-3’
	Reverse：5’-CCTGAGCAAGGATATTCACGACA-3’
Human MVK primer	Forward：5’-TGCTGGAAGAGCTCATTGACAT-3’
	Reverse：5’-GTTTCCAAGCAGTCAAAGCCA-3’
Human MVD primer	Forward：5’-ATGCCGTGATCTTCACCCTG-3’
	Reverse：5’-TGTATTTGACCCCACCGGG-3’
Human LSS primer	Forward：5’-ATAACACATGCTGGGCCATGA-3’
	Reverse：5’-AGCTCGTGTAGGAGATGGCA-3’
Human DHCR24 primer	Forward：5’-TGGAGGCTATCTGTGCCAAG-3’

	Reverse：	5’-ATGTACTCCAGGCCCTCTCG-3’
Human DHCR7 primer	Forward：	5’-ACACGCTGCAGGGTCTGTA-3’
	Reverse：	5’-CACTCGATGACCTTGGGCTT-3’
Human IDI1 primer	Forward：	5’-TAAGCAATCCAGCCGAGCTT-3’
	Reverse：	5’-TCACCCCAGATACCATCAGACT-3’
Human CYP51A1 primer	Forward：	5’-GGGTAGAACGCCTGGACTTT-3’
	Reverse：	5’-CTGGGTTTTCAGGGGTGTGAA-3’
Human PMVK primer	Forward：	5’-CCTTTCGGAAGGACATGATCC-3’
	Reverse：	5’-TCTCCGTGTGTCACTCACCA-3’
Human HBA primer	Forward：	5’-GCTCTGCCCAGGTTAAGGG-3’
	Reverse：	5’-CAGTGGCTTAGGAGCTTGAAG-3’
Human HBB primer	Forward：	5’-CTTTGCCACACTGAGTGAGCTG-3’
	Reverse：	5’-CACCAGCCACCACTTTCTGAT-3’
Human ABCG1 primer	Forward：	5’-TCTTCGTCAGCTTCGACACC-3’
	Reverse：	5’-GTCCAGGTACAGCTTGGCAT-3’
Human ABCA1 primer	Forward：	5’-CTCTGGCCAGGATATTCAGCA-3’
	Reverse：	5’-GATTGCCAAAACGGTCACCAG-3’
Human GAPDH primer	Forward：	5’-GGAGTCCACTGGCGTCTTCAC-3’
	Reverse：	5’-GAGGCATTGCTGATGATCTTGAG-3’
Human ACAT1 primer	Forward：	5’-AAGGCAGGCAGTATTGGGTG-3’
	Reverse：	5’-ACATCAGTTAGCCCGTCTTTTAC-3’
Human ACAT2 primer	Forward：	5’-GCGGACCATCATAGGTTCCTT-3’
	Reverse：	5’-ACTGGCTTGTCTAACAGGATTCT-3’
Human HMGCR primer	Forward：	5’-AATCCTGGGGAAAATGCCCG-3’
	Reverse：	5’-GCTGTCTTCTTGGTGCAAGC-3’
Human HMGCS1 primer	Forward：	5’-CAGAAGAACTTACGCTCGGC-3’
	Reverse：	5’-GGTACTTTCTTGGCAGGGCT-3’
Human SREBP2 primer	Forward：	5’-CCTGGGAGACATCGACGAGAT-3’
	Reverse：	5’-TGAATGACCGTTGCACTGAAG-3’
Mouse HMGCR primer	Forward：	5’-GCTGTCTTCTTGGTGCAAGC-3’
	Reverse：	5’-AACGGTCTCCCTAACAACCG-3’

Mouse HMGCS1 primer	Forward：	5’-AACTGGTGCAGAAATCTCTAGC-3’
	Reverse：	5’-GGTTGAATAGCTCAGAACTAGCC-3’
Mouse SREBP2 primer	Forward：	5’-GCAGCAACGGGACCATTCT-3’
	Reverse：	5’-CCCCATGACTAAGTCCTTCAACT-3’
Mouse MVK primer	Forward：	5’-GGTGTGGTCGGAACTTCCC-3’
	Reverse：	5’-CCTTGAGCGGGTTGGAGAC-3’
Mouse FDFT1 primer	Forward：	5’-ATGGAGTTCGTCAAGTGTCTAGG-3’
	Reverse：	5’-CGTGCCGTATGTCCCCATC-3’
Mouse SQLE primer	Forward：	5’-ATAAGAAATGCGGGGATGTCAC-3’
	Reverse：	5’-ATATCCGAGAAGGCAGCGAAC-3’
Mouse LSS primer	Forward：	5’-TCGTGGGGGACCCTATAAAAC-3’
	Reverse：	5’-CGTCCTCCGCTTGATAATAAGTC-3’
Mouse DHCR24 primer	Forward：	5’-CTCTGGGTGCGAGTGAAGG-3’
	Reverse：	5’-TTCCCGGACCTGTTTCTGGAT-3’
Mouse IDI1 primer	Forward：	5’-ACCAGCCATCTTGATGAAAAACA-3’
	Reverse：	5’-CAGCAACTATTGGTGAAACAACC-3’
Mouse MVD primer	Forward：	5’-ATGGCCTCAGAAAAGCCTCAG-3’
	Reverse：	5’- TGGTCGTTTTTAGCTGGTCCT-3’
Mouse FDPS primer	Forward：	5’-GGAGGTCCTAGAGTACAATGCC-3’
	Reverse：	5’-AAGCCTGGAGCAGTTCTACAC-3’
Mouse CYP51A1 primer	Forward：	5’-GACAGGAGGCAACTTGCTTTC-3’
	Reverse：	5’-GTGGACTTTTCGCTCCAGC-3’
Mouse DHCR7 primer	Forward：	5’-AGGCTGGATCTCAAGGACAAT-3’
	Reverse：	5’-GCCAGACTAGCATGGCCTG-3’
Mouse 18sRNA primer	Forward：	5’-GCAATTATTCCCCATGAACG-3’
	Reverse：	5’-GGCCTCACTAAACCATCCAA-3’
Mouse LDLR primer	Forward：	5’-ATCTAGGCAATCTCGGTCTCC-3’
	Reverse：	5’-TGACTCAGACGAACAAGGCTG-3’
Mouse MVK primer	Forward：	5’-GGTGTGGTCGGAACTTCCC-3’
	Reverse：	5’-CCTTGAGCGGGTTGGAGAC-3’
Mouse ACAT1 primer	Forward：	5’-CAGGAAGTAAGATGCCTGGAAC-3’

	Reverse：5’-TTCACCCCCTTGGATGACATT-3’
Mouse ACAT2 primer	Forward：5’-CCCGTGGTCATCGTCTCAG-3’
	Reverse：5’-GGACAGGGCACCATTGAAGG-3’
Mouse ABCA1 primer	Forward：5’-AAAACCGCAGACATCCTTCAG-3’
	Reverse：5’-CATACCGAAACTCGTTCACCC-3’
Mouse ABCG1 primer	Forward：5’-CTTTCCTACTCTGTACCCGAGG-3’
	Reverse：5’-CGGGGCATTCCATTGATAAGG-3’
Mouse HBA-A1 primer	Forward：5’-CACCACCAAGACCTACTTCC-3’
	Reverse：5’-CAGTGGCTCAGGAGCTTGA-3’
Mouse HBB-B1 primer	Forward：5’-GCACCTGACTGATGCTGAGAA-3’
	Reverse：5’-TTCATCGGAGTTCACCTTTCC-3’
Mouse GATA-1primer	Forward：5’-TGGGGACCTCAGAACCCTTG-3’
	Reverse：5’-GGCTGCATTTGGGGAAGTG-3’
Mouse NF-E2 primer	Forward：5’-TCCTCAGCAGAACAGGAACAG-3’
	Reverse：5’-GGCTCAAAAGATGTCTCACTTGG-3’
Mouse -NF-E2-shRNA-1	TTTGGCCCATCCCAACTATAC
Mouse -NFE2-shRNA-2	TCTGATGAAGGCACTTCTTAA
Mouse -GATA1-shRNA-1	ACTGAGATTCAGGCATGTATT
Mouse -GATA1-shRNA-2	GCTCAACAGTATGGAGGGAAT
Mouse -SREBP2-shRNA-1	GCGGACAACACACAATATCAT
Mouse -SREBP2-shRNA-2	TTAACACTGCTGTGGTAAATC
Human-SREBP2-shRNA-1	GCCCTCTATTGGATGATGCAA
Human-SREBP2-shRNA-3	CCTGAGTTTCTCTCTCCTGAA
