# Supplementary material for: Fine‐Tuning of Cholesterol Homeostasis Controls Erythroid Differentiation
Source: Adv Sci (Weinh). 2021 Nov 5;9(2):2102669. doi: 10.1002/advs.202102669 (PMC8805577; doi:10.1002/advs.202102669)
Supplement: Supplementary file 2 — Supplemental Figure 1 [file ADVS-9-2102669-s002.pdf]

Fig. S1 supplemental

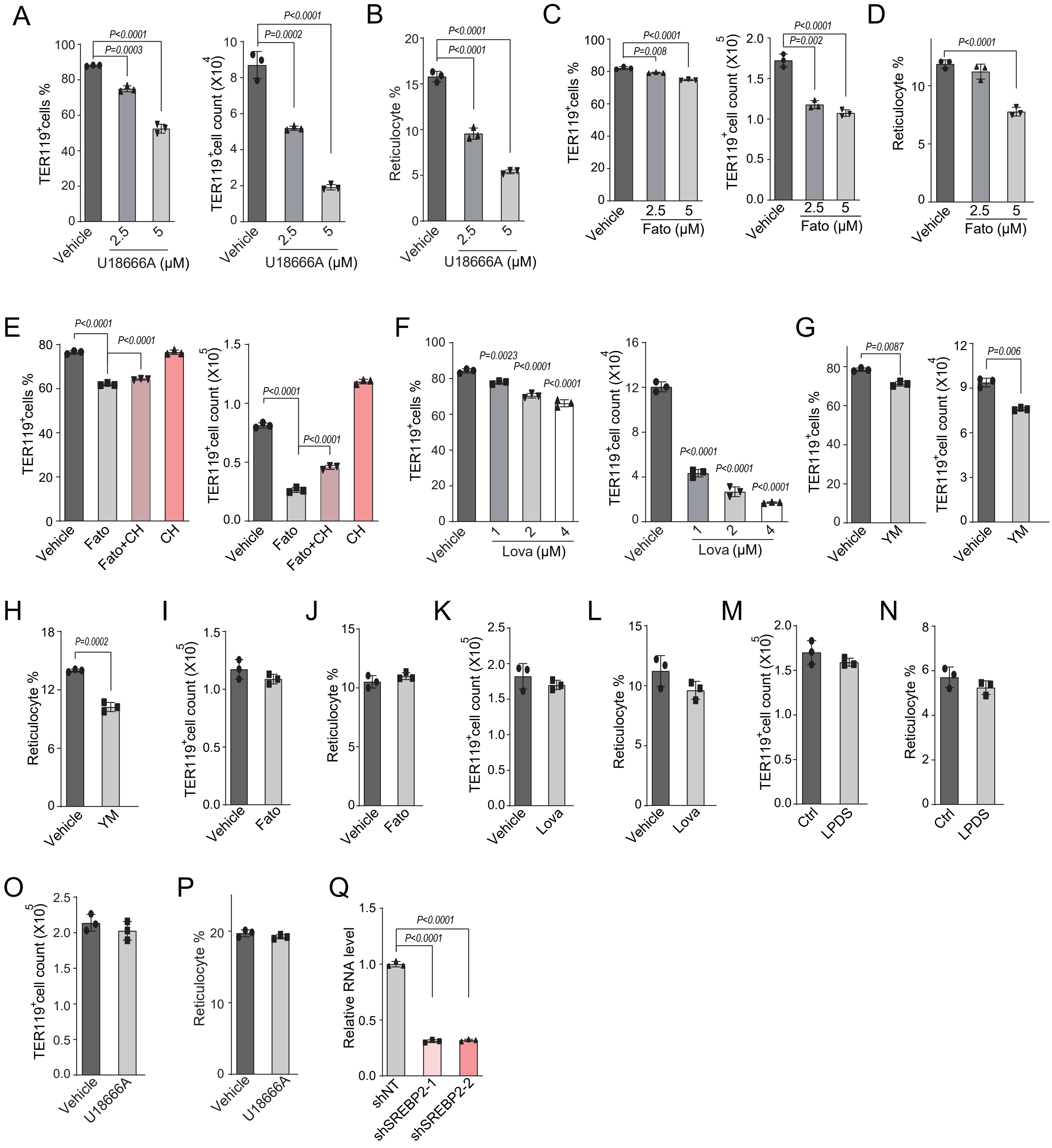

Figure S1 Inhibition of cholesterol biosynthesis affects normal terminal erythroid differentiation, related to Figure 1.
(A) Quantification of percentage (left) and cell number of $TER119^+$ cells (right) in differentiated erythroblasts after treatment with U18666A for 24 h. FLCs were purified and cultured in Epo containing medium. (B) Statistical analysis of the percentage of reticulocytes after treatment with U18666A for 48 h. FLCs were purified and cultured in Epo containing medium. (C) Quantificationof percentage (left) and cell number of $TER119^+$ cells (right) in differentiated erythroblasts after treatment with fatostatin for 24 h. (D) Statistical analysis of reticulocytes after FLCs were treated with fatostatin for 48 h. (E) Quantification of percentage (left) and cell number of $TER119^+$ cells (right) in differentiated erythroblasts after treatment with indicated compounds for 24 h. FLCs were cultured as in (A). CH indicated cholesterol. (F) FLCs were cultured with or without indicated dose of lovastatin for 24 h, and percentage (left) and number of $TER119^+$ cell (right) was measured by flow cytometry. (G-H) FLCs were cultured in Epo containing medium and treated with YM53601. Percentage and cell number of $TER119^+$ cells (G) were measured by flow cytometry at 24 h. Percentage of reticulocytes (H) was measured by flow cytometry at 48 h using TER119 and Hoechst staining. (I-P) FLCs were cultured in Epo containing medium for 30 h, then cells were treated with fatostatin (I-G), lovastatin (K-L), Lipoprotein deficient serum (LPDS, M-N), or U18666A (O-P) for additional 18 h, cell number of $TER119^+$ cell and percentage of reticulocytes were analyzed by flow cytometry. (Q) Quantification of the mRNA expression of *SREBP2* in cells from Figure 1G. *P* values were determined by using unpaired two-tailed Student’s t-test (G, H) or 1-way ANOVA with Tukey’s multiple comparisons test (A-F, Q). Data are presented as mean ± SD from three independent experiments.

Fig. S2 supplemental

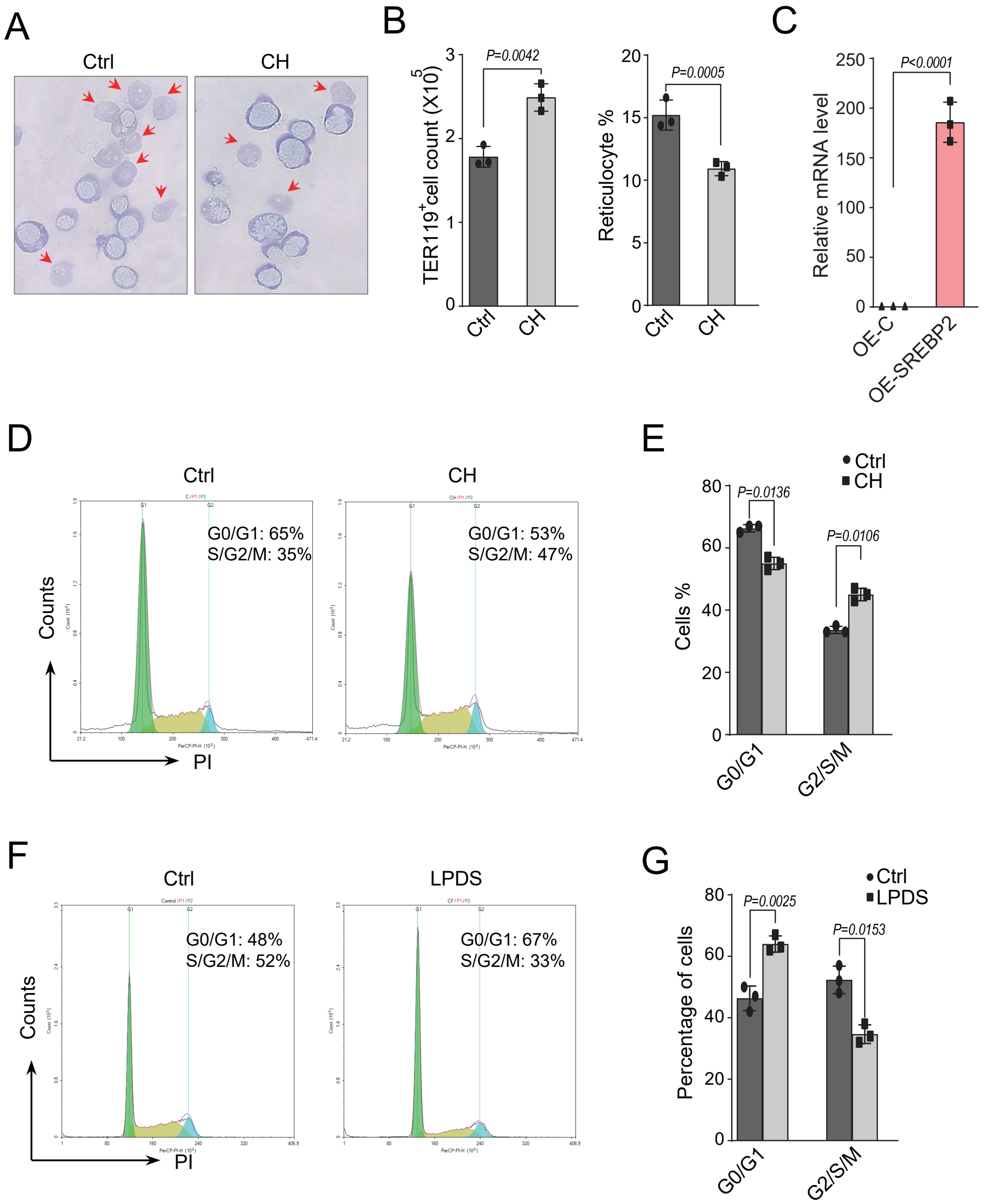

Figure S2 Excess cholesterol disrupts normal cell cycle process and erythroid differentiation in the late stages of terminal erythropoiesis, related to Figure 2.

(A) Morphologic analysis of erythroid differentiation after in vitro culture for 48 h by benzidine-Giemsa staining. Ctrl: control; CH: cholesterol. Arrows indicated reticulocytes. (B) FLCs were cultured in Epo medium for 30 h, and then treated with or without cholesterol (40 μM) for additional 18 h. Cell number of $TER119^{+}$ cells and percentage of reticulocytes were analyzed by flow cytometry using TER119 and Hoechst staining. (C) Quantification of the mRNA expression of human *SREBP2* in cells from Figure 2E. (D) Representative cell cycle profiles of erythroblasts after 30 h of *in vitro* culture with or without cholesterol (40 μM). Cell cycle was analyzed by flow cytometry using propidium iodide staining. Percentage of cells in indicated phases is shown. (E) Quantification of cells in indicated phases from (D). (F) Representative cell cycle profiles of erythroblasts after 30 h of *in vitro* culture with normal FBS (Ctrl) or lipoprotein deficient serum (LPDS). (G) Quantification of cells in indicated phases from (F). All *P* values were determined by unpaired two-tailed Student's t-test. Data are presented as mean ± SD from three independent experiments.

Fig. S3 supplemental

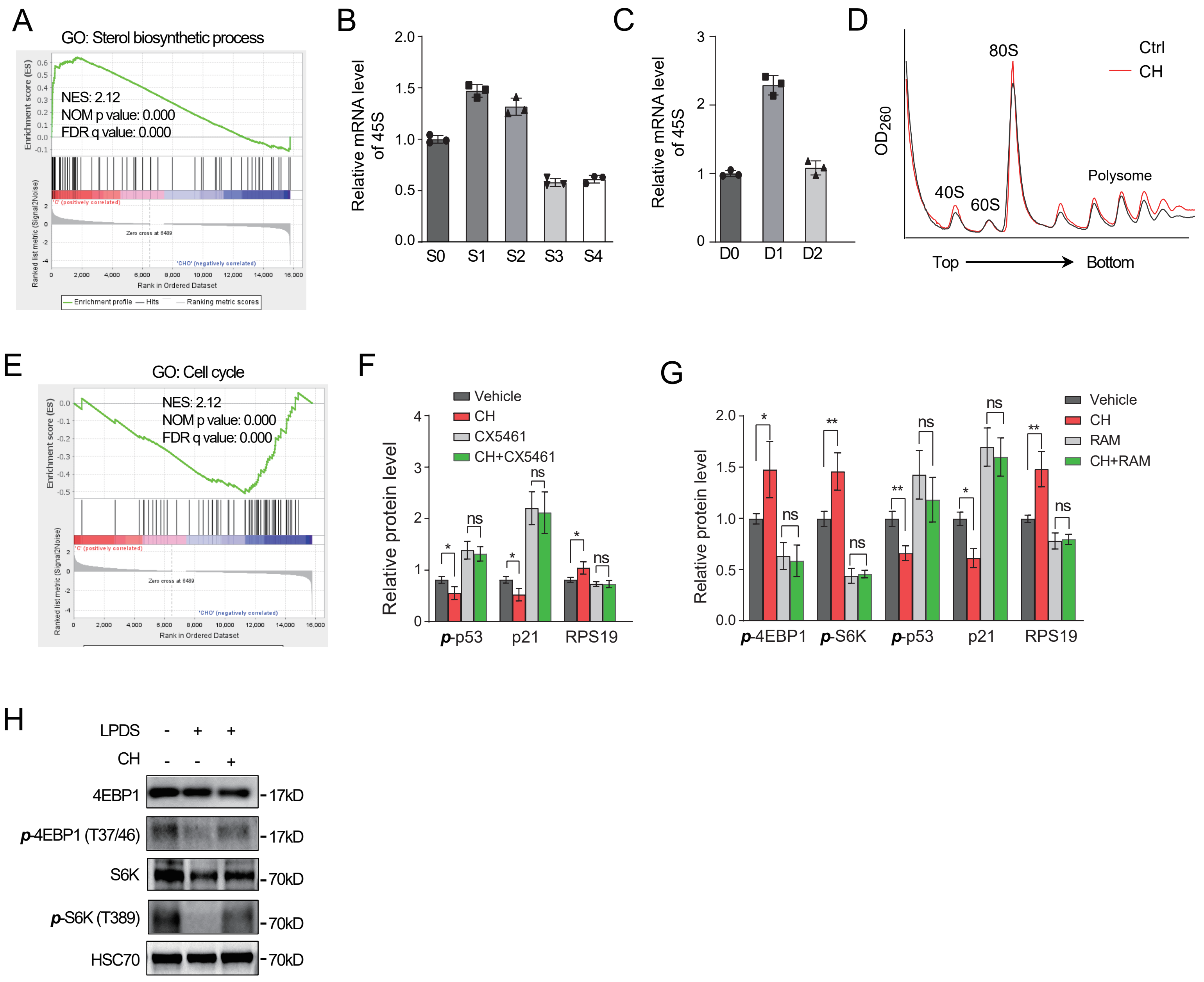

Figure S3 Ribosome biogenesis is downregulated during terminal erythropoiesis, related to Figure 3.

(A) Gene Set Enrichment Analysis of sterol biosynthetic pathway in erythroblasts treated by cholesterol (CH, 40 µM) or vehicle (control, Ctrl). Cell was treated as in Figure 3A. (B) Quantification of pre-rRNA 45S transcripts in E14.5 mouse fetal liver cells shown as in Figure 4D. (C) Quantification of pre-rRNA 45S transcripts in cultured erythroblasts. D0 to D2 indicate different days of TER119 negative mouse fetal liver erythroblasts *in vitro* culture. (D) Polysome profiling from cells treated as in Figure 3C. (E) Gene Set Enrichment Analysis of cell cycle pathway in erythroblasts treated by cholesterol (CH, 40 µM) or vehicle (control, Ctrl). Cell was treated as in Figure 3A. (F) Quantification of relative protein levels of Figure 3I. (G) Quantification of relative protein levels of Figure 3J. (H) Western blot analysis of indicated proteins in erythroblasts after 30 h of *in vitro* culture with lipoprotein deficient serum (LPDS) or plus with cholesterol (CH, 40 µM). HSC70 was used as a loading control. An equal number of cells were loaded in each well. All *P* values were determined by unpaired two-tailed Student's t-test. Data are presented as mean ± SD from three independent experiments.

Fig. S4 supplemental

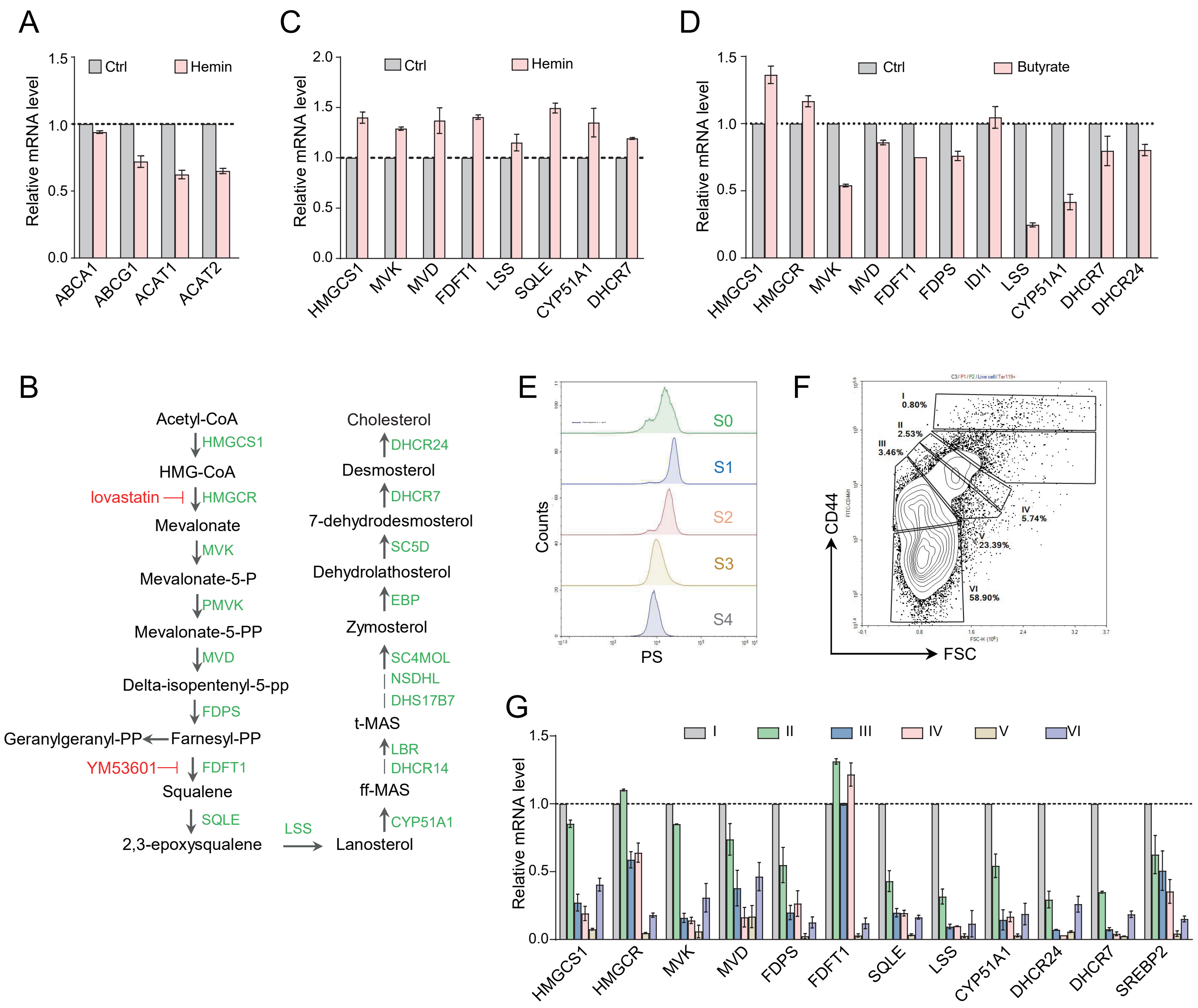

Figure S4 Cholesterol synthesis is down-regulated during terminal erythroid differentiation, related to Figure 4.

(A) Quantification of the mRNA level of indicated genes in K562 cells after incubation of hemin for 24 h. (B) Schematic diagram of cholesterol biosynthesis pathway. Cholesterologenic enzymes and relative inhibitors are highlighted in green and red respectively. (C) Quantification of the mRNA level of indicated genes in Hela cells after incubation of hemin (40 μM) for 24 h. (D) Quantification of the mRNA level of genes encoding cholesterologenic enzymes in K562 cells after incubation of butyrate (1 mM) for 48 h. (E) Representative flow cytometric profiles of cellular phosphatidylserine in indicated cells as in Figure 4D using annexin V staining. (F) Representative flow cytometric profiles of erythroid populations at different developmental stages in bone marrow. Cells were gated based on cell size and CD44 expression. Populations I to VI represent the least differentiated to enucleated RBC. (G) Quantification of the mRNA expression of indicated genes in cells sorted according to (F). Transcripts were normalized to 18S ribosomal RNA. Data are presented as mean ± SD from three independent experiments.

Fig. S5 supplemental

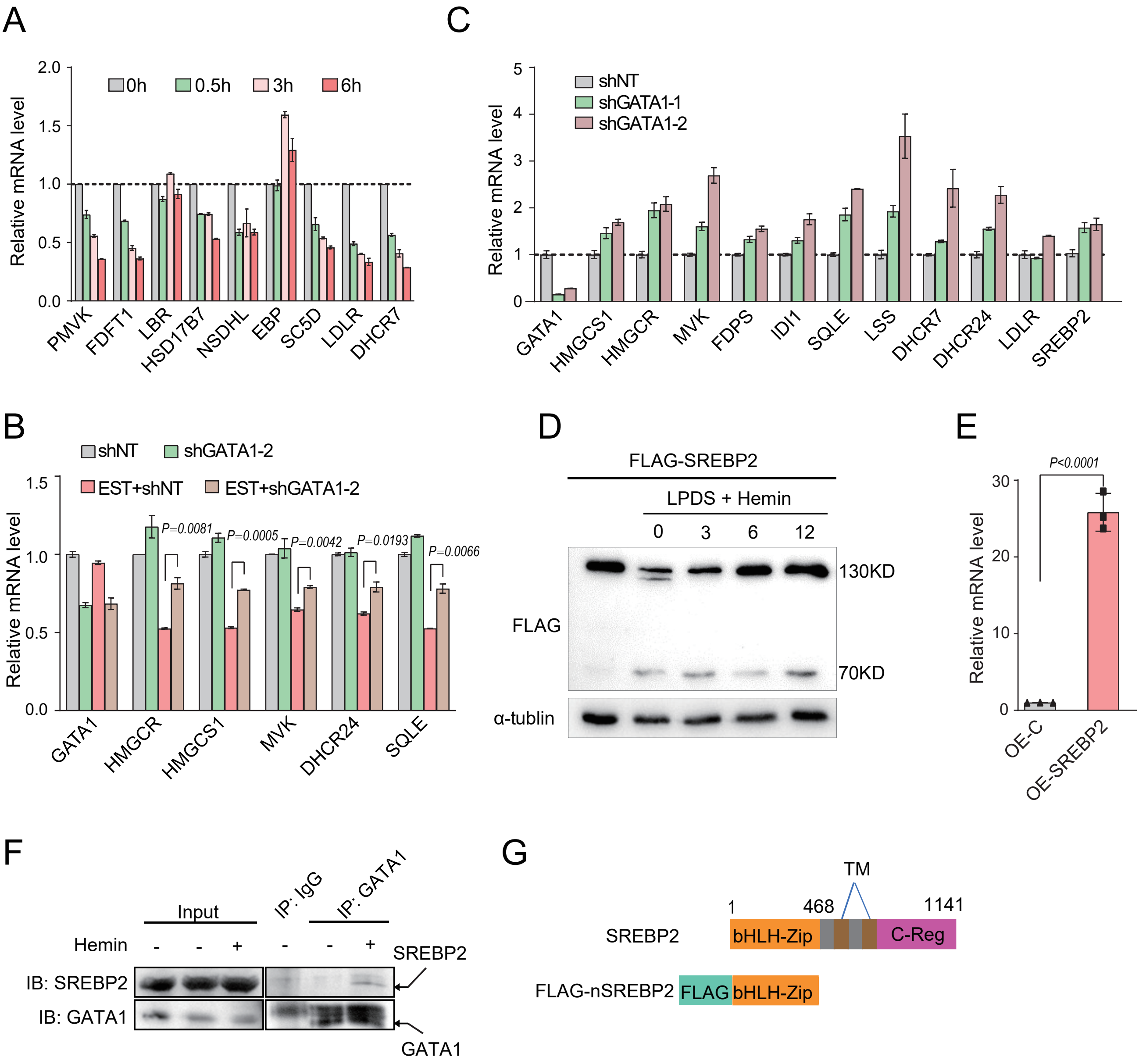

Figure S5 GATA1 downregulates cholesterol biosynthesis, related to Figure 5.

(A) Quantification of indicated genes in G1-ER4 cells after induction of β-estradiol (EST, 500 nM). Data are presented as mean ± SD from three independent experiments. (B) Quantification of indicated genes in G1-ER4 cells with GATA1 knockdown. shGATA1-2 represented a different shRNA targeting GATA1 with the one used in Figure 5B. (C) K562 cells were transduced with lentivirus encoding GATA1 shRNA and the mRNA expression of *GATA1* and cholesterol synthesis related genes were analyzed by quantitative PCR. (D) Immunoblot analysis of SREBP2 activation in K562 cells. K562 cells stably expressing FLAG-nSREBP2 were cultured with normal FBS or lipoprotein deficient serum (LPDS) supplemented with 5 μM lovastatin, and then incubated with 40 μM hemin or vehicle for indicated time. (E) Quantification of the mRNA expression of human SREBP2 in cells from Figure 5C. (F) Co-IP of endogenous SREBP2 and GATA1 in K562 cells with or without incubation of hemin. (G) Structure of full-length and active SREBP2. TM: transmembrane; bHLH-Zip: basic helix-loop-helix; C-reg: c-regulatory. Transcripts were normalized to 18S ribosomal RNA. *P* value was determined by unpaired two-tailed Student's t-test. Data are presented as mean ± SD from three independent experiments.

Fig. S6 supplemental

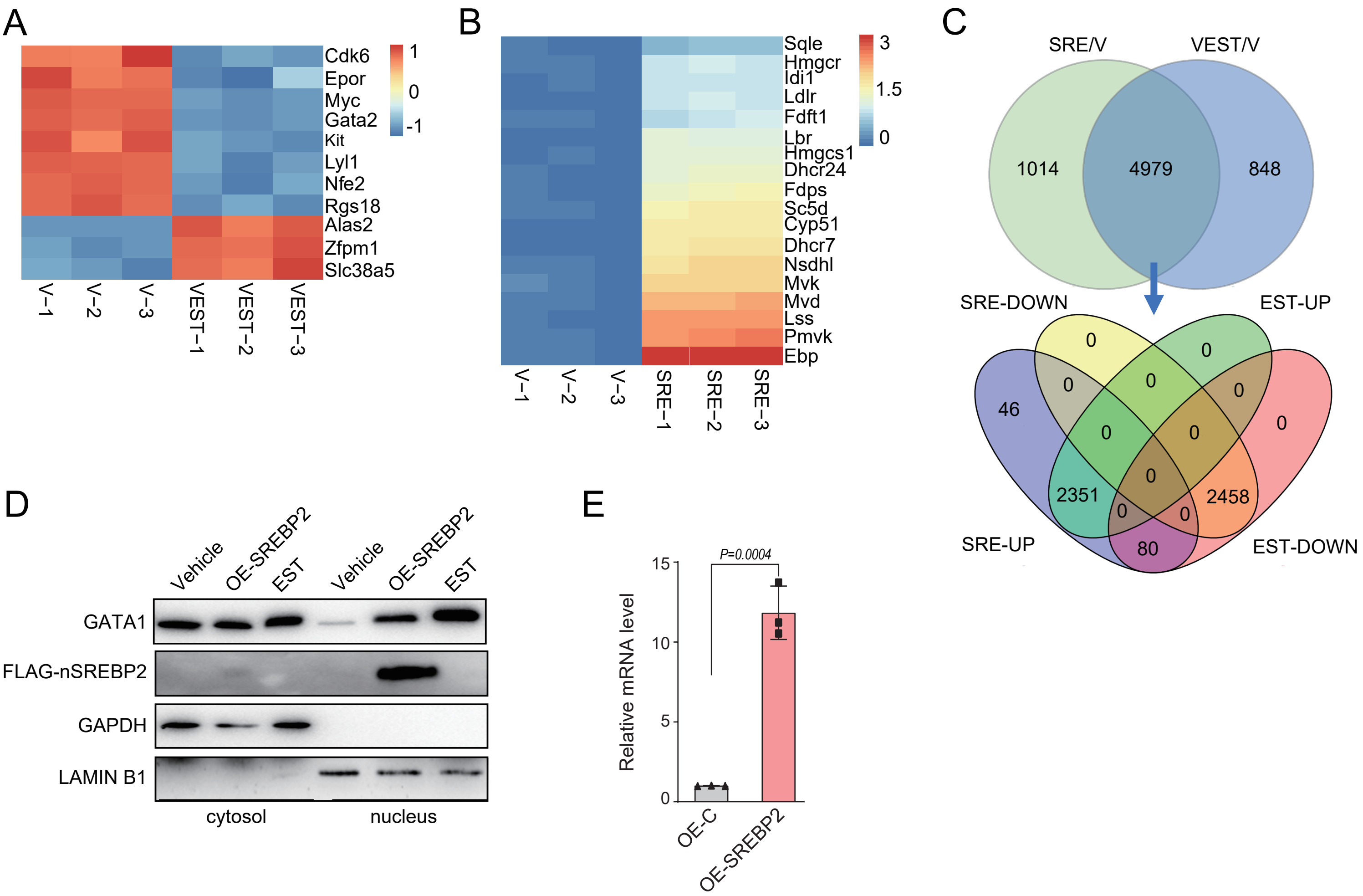

Figure S6 Transcriptional control of NFE2 by SREBP2 contributes to the regulation of globin expression GATA1 downregulates cholesterol biosynthesis, related to Figure 6.

(A) A heatmap of representative GATA1 target genes with or without EST stimulation (n=3, adjusted p value [p-adj] < 0.05). (B) A heatmap of the genes encoding cholesterologenic enzymes upregulated by SREBP2 overexpression (n=3, adjusted p value [p-adj] < 0.05). (C) Venn diagram indicated the number of differential genes and common genes between SREBP2 overexpression and EST treatment (1.2-fold change cutoff). (D) Immunoblot analysis of nuclear localization of the GATA1/ER proteins induced by SREBP2 overexpression or EST. GAPDH and Lamin B1 were the endogenous markers for cytosolic and nuclear proteins. (E) Quantification of the mRNA expression of human *SREBP2* in cells from Figure 6C. Transcripts were normalized to 18S ribosomal RNA. *P* value was determined by unpaired two-tailed Student's t-test. Data are presented as mean ± SD from three independent experiments.

Fig. S7 supplemental

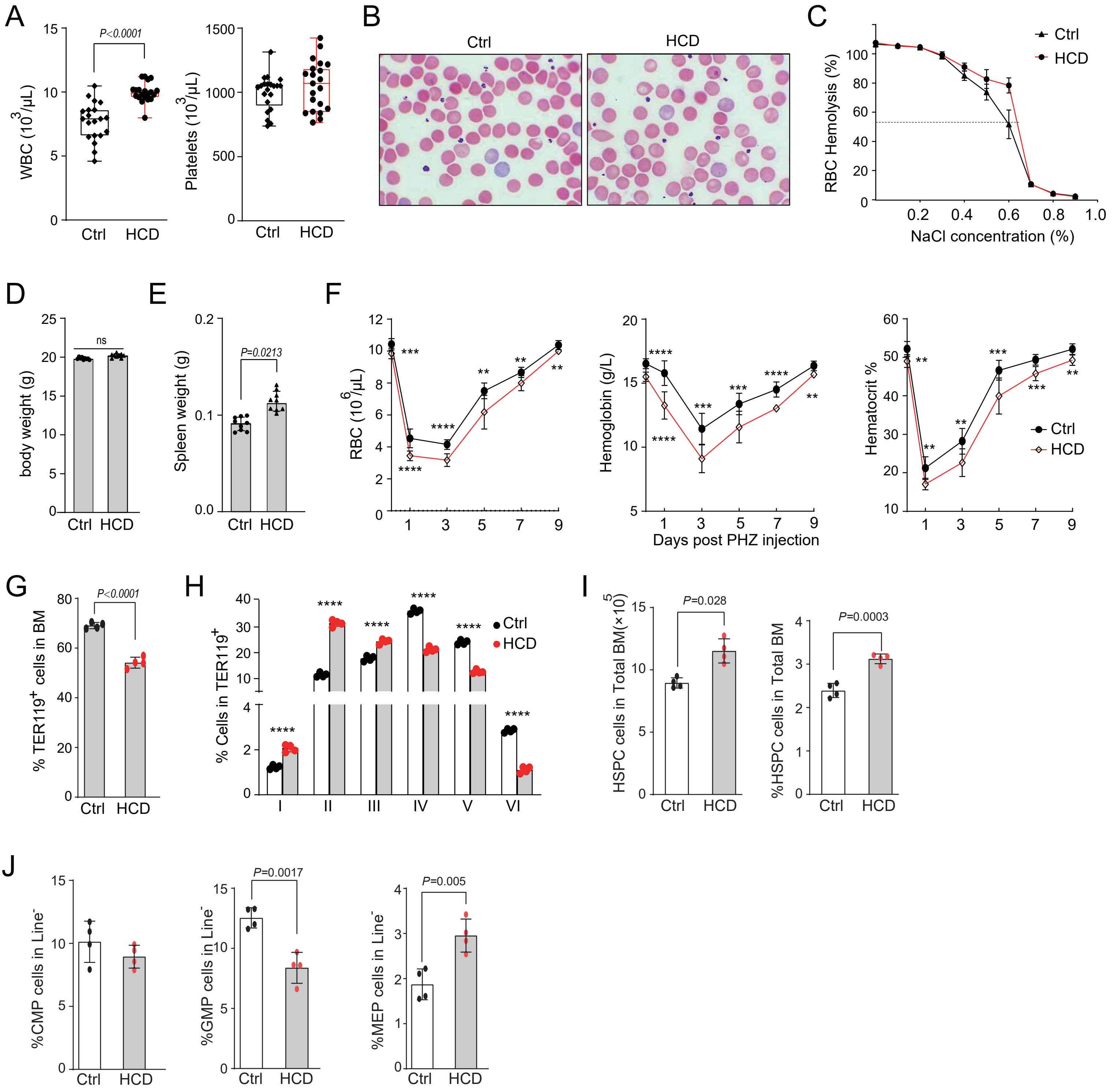

Figure S7 Excess cholesterol impairs erythropoiesis in vivo, related to Figure 7.

(A) White blood cells (WBC) and platelets indices of indicated mice. C57BL/6J mice were fed on chow diets (n=9) or high cholesterol diets (HCD) (n=9) for 4 weeks. Both males and females were included in each group. (B) Representative morphologic analysis of peripheral blood smear from (A) by benzidine-Giemsa staining. (C) The osmotic fragility and hemolysis of red blood cells from indicated mice from (A). (D-E) Statistical analysis of body and spleen weight of indicated mice from (A). (F) Indicated mice from (A) were injected with PHZ on day 0. Red blood cells (RBC), hemoglobin and hematocrit indices were measured on indicated days afterPHZ injection. Data are presented as mean ± SD from 9 mice for each group. $**P < 0.01$, $***P < 0.001$ and $****P < 0.0001$. (G) Quantification of TER119$^+$ cells in bone marrow from indicated mice on day 9 after PHZ injection. Each dot represents one mouse. (H) Quantification of indicated populations from (G). Populations I to VI represent the least differentiated to enucleated RBC as shown as in Figure 7D. Data are presented as mean ± SD from 4 mice for each group. $****P < 0.0001$. (I) Quantification of percentage and cell number of hematopoietic stem and progenitor cells (HSPCs) in HCD mice bone marrow from Figure 7E. Each dot represents one mouse. (J) Quantification of indicated populations in HSPC cells from indicated mice bone marrow from (H). HSPCs are divided into three subpopulations which are common myeloid progenitors (CMPs), granulocyte-macrophage progenitors (GMPs) and megakaryocyte-erythrocyte progenitors (MEPs) according to CD16/32 and CD34. Each dot represents one mouse. Data are presented as mean ± SD from 4 mice for each group. *P* values were determined by using unpaired two-tailed Student's t-test (A, E, G, I, G, H) or 1-way ANOVA with Tukey's multiple comparisons test (F).

Fig. S8 supplemental

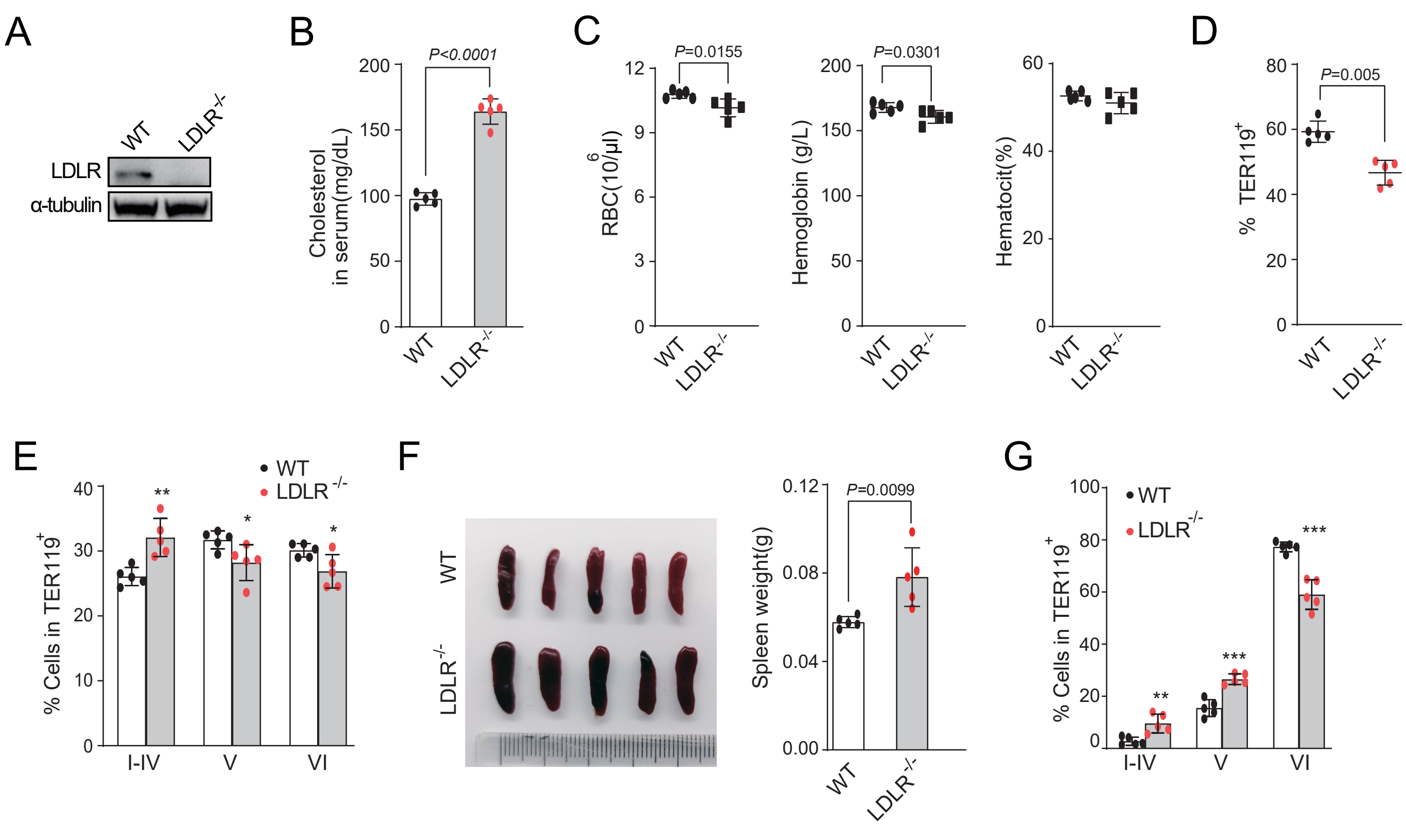

Figure S8 Loss of LDLR impairs erythropoiesis *in vivo*, related to Figure 7.

(A) Western blot analysis of LDLR proteins in erythroblasts from indicated mice bone marrow. α-tubulin was used as a loading control. An equal number of cells were loaded in each well. (B) Quantification of serum cholesterol in LDLR knockout mice. Each dot represents one mouse. Five males at the age of 8 weeks were included in each group. (C) Red blood cells (RBC), hemoglobin and hematocrit indices of indicated mice from (B). (D) Quantification of percentage of TER119$^+$ cells in bone marrow of indicated mice from (B). (E) Quantification of indicated populations in TER119$^+$ cells from (D). Populations I to VI represent the least differentiated to enucleated RBC. (F) Photomicrographs of spleens and statistical analysis of the spleen weight from indicated mice are shown in (B). (G) Quantification of indicated populations in TER119$^+$ cells from indicated mice spleen in (F). All *P* values were determined by unpaired two-tailed Student's t-test. Data are presented as mean ± SD from 5 mice for each group.

Fig. S9 supplemental

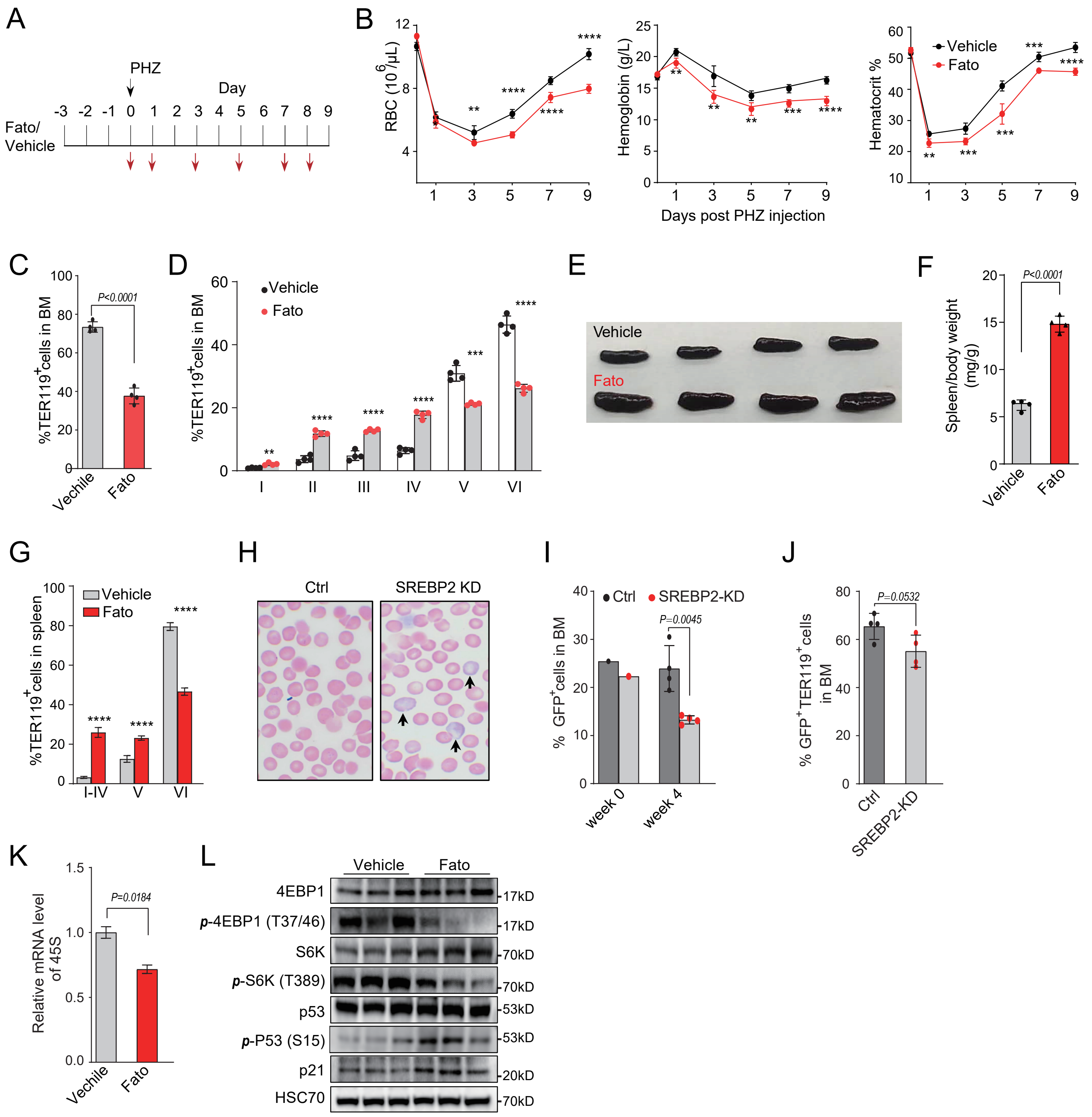

Figure S9 Disruption of cholesterol synthesis impairs normal erythropoiesis in vivo, related to Figure 8.
(A) Experimental design for fatostatin-treated mice in response to PHZ-induced hemolytic anemia. Red arrows indicate the timepoint for complete blood count test. All drugs were administrated by intraperitoneal injection. Both males and females were included in each group. (B) Red blood cells (RBC), hemoglobin and hematocrit indices of indicated mice in (A). Each dot represents one mouse. Data are presented as mean ± SD from 6 mice for each group. (C) Quantification of TER119+ cells in bone marrow from indicated mice onday 9 after PHZ injection. Data are presented as mean ± SD from 4 mice for each group. (D) Quantification of indicated populations in $TER119^+$ erythroid cells from indicated mice bone marrow in (C). Populations I to VI represent the least differentiated to enucleated RBC as shown as in Figure 7D. Data are presented as mean ± SD from 4 mice for each group. $^{**}P < 0.01$, $^{***}P < 0.001$ and $^{****}P < 0.0001$. (E) Photomicrographs of spleens from indicated mice as in (C). (F) Statistical analysis of the spleen-to-body-weight ratio (Vehicle, n=4; fatostatin, n=4). (G) Quantification of indicated populations in $TER119^+$ erythroid cells from indicated mice spleen in (C). (H) Representative morphologic analysis of peripheral blood smear from indicated mice as in Figure 8A by benzidine-Giemsa staining. (I) Quantification of percentage of GFP^+ cells in indicated groups before and in bone marrow cells after 4 weeks transplantation respectively. (J) Quantification of percentage of $GFP^+TER119^+$ cells in bone marrow from indicated mice at 4 weeks after transplantation. Data are presented as mean ± SD from 4 mice for each group. (K) Quantification of transcripts of pre-rRNA 45S in erythroblasts from indicated mice bone marrow as in (C). (L) Western blot analysis of indicated proteins in erythroblasts from indicated mice bone marrow as in (C). HSC70 was used as a loading control. An equal number of cells were loaded in each well. All *P* values were determined by unpaired two-tailed Student's t-test.

Fig. S10 supplemental

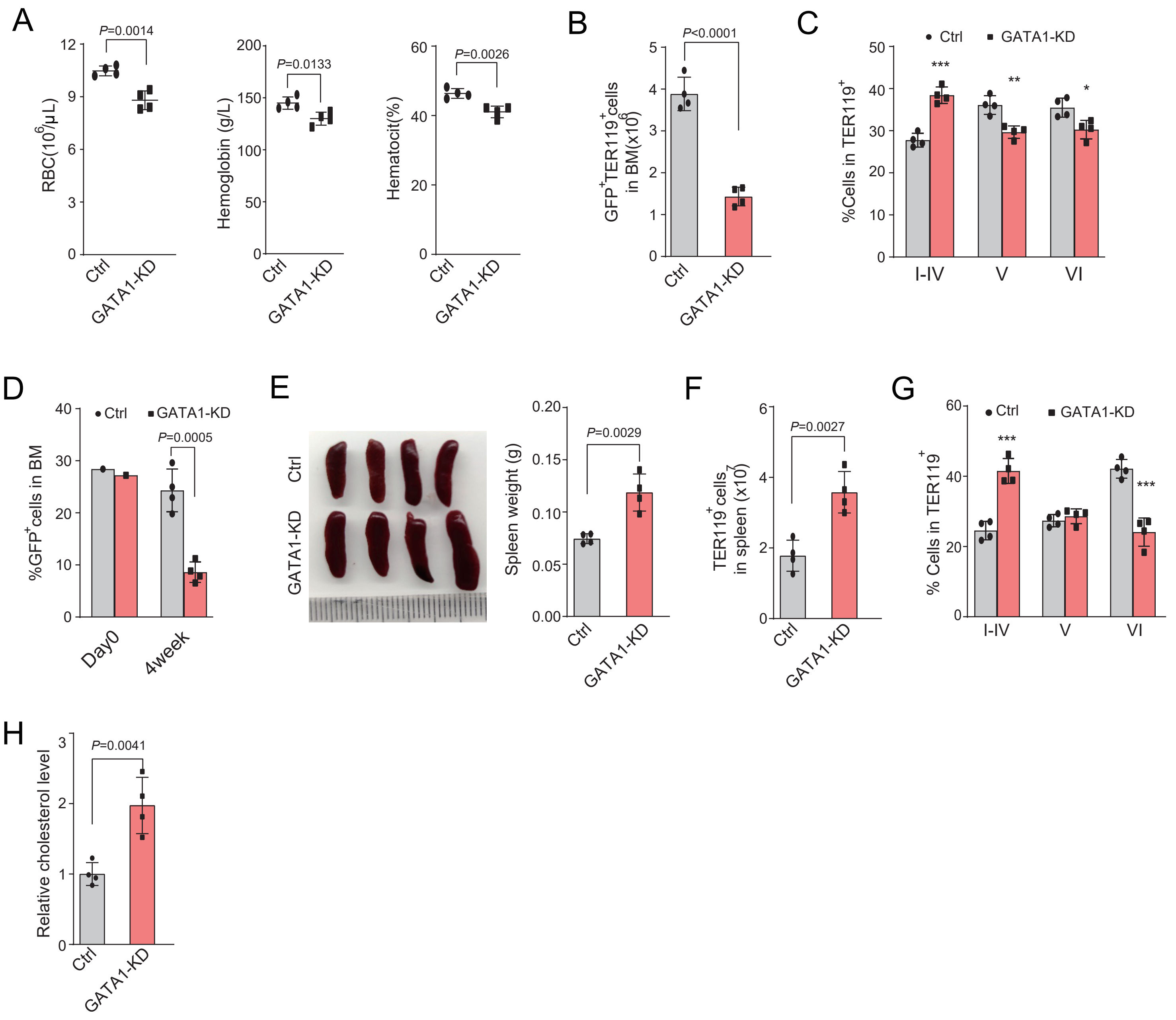

Figure S10 Knockdown GATA1 promotes cholesterol synthesis *in vivo*, related to Figure 8.

(A) Red blood cells (RBC), hemoglobin and hematocrit indices of indicated mice at 4 weeks after transplantation (Control, n=4; GATA1-KD, n=4). Each dot represents one mouse. (B) Quantification of cell number of $GFP^{+}TER119^{+}$ cells in bone marrow from indicated mice in (A). (C) Quantification of indicated populations in TER119+ cells from indicated mice bone marrow in (B). (D) Quantification of percentage of GFP^{+} cells in indicated groups before and in bone marrow cells after 4 weeks transplantation respectively. (E) Photomicrographs of spleens and statistical analysis of the spleen weight from indicated mice are shown in (B). (F) Quantification of percentage of $TER119^{+}$ cells in spleen from indicated mice. (G) Quantification of indicated populations in $TER119^{+}$ erythroid cells from indicated mice spleen in (F). (H) Intracellular cholesterol levels were analyzed by flow cytometry based on the intensity of Filipin III staining of $TER119^{+}$ cells from indicated mice in (B). **P* < 0.05, ***P* < 0.01 and ****P* < 0.001. All *P* values were determined by unpaired two-tailed Student's t-test. Data are presented as mean ± SD from 4 mice for each group.
